# VCP suppresses proteopathic seeding in neurons

**DOI:** 10.1186/s13024-022-00532-0

**Published:** 2022-04-12

**Authors:** Jiang Zhu, Sara Pittman, Dhruva Dhavale, Rachel French, Jessica N. Patterson, Mohamed Salman Kaleelurrrahuman, Yuanzi Sun, Jaime Vaquer-Alicea, Gianna Maggiore, Christoph S. Clemen, William J. Buscher, Jan Bieschke, Paul Kotzbauer, Yuna Ayala, Marc I. Diamond, Albert A. Davis, Conrad Weihl

**Affiliations:** 1 Department of Neurology, Hope Center for Neurological Diseases, Washington University School of Medicine, St Louis, MO 63110 USA; 2 Edward A. Doisy Department of Biochemistry and Molecular Biology, Saint Louis University School of Medicine, St. Louis, MO USA; 3 Medical Research Council Prion Unit / UCL Institute of Prion Diseases, University College London, London, UK; 4 Center for Alzheimer’s and Neurodegenerative Diseases, Peter O’ Donnell Jr. Brain Institute, University of Texas Southwestern Medical Center, Dallas, TX USA; 5 Institute of Aerospace Medicine, German Aerospace Center, Cologne, Germany; 6 Center for Physiology and Pathophysiology, Institute of Vegetative Physiology, Medical Faculty, University of Cologne, Cologne, Germany; 7 Department of Genetics, Washington University School of Medicine, St. Louis, MO USA

**Keywords:** CRISPR screen, Seeding, Alpha-synuclein, TDP-43, Frontotemporal dementia

## Abstract

**Background:**

Neuronal uptake and subsequent spread of proteopathic seeds, such as αS (alpha-synuclein), Tau, and TDP-43, contribute to neurodegeneration. The cellular machinery participating in this process is poorly understood. One proteinopathy called multisystem proteinopathy (MSP) is associated with dominant mutations in Valosin Containing Protein (VCP). MSP patients have muscle and neuronal degeneration characterized by aggregate pathology that can include αS, Tau and TDP-43.

**Methods:**

We performed a fluorescent cell sorting based genome-wide CRISPR-Cas9 screen in αS biosensors. αS and TDP-43 seeding activity under varied conditions was assessed using FRET/Flow biosensor cells or immunofluorescence for phosphorylated αS or TDP-43 in primary cultured neurons. We analyzed in vivo seeding activity by immunostaining for phosphorylated αS following intrastriatal injection of αS seeds in control or VCP disease mutation carrying mice.

**Results:**

One hundred fifty-four genes were identified as suppressors of αS seeding. One suppressor, VCP when chemically or genetically inhibited increased αS seeding in cells and neurons. This was not due to an increase in αS uptake or αS protein levels. MSP-VCP mutation expression increased αS seeding in cells and neurons. Intrastriatal injection of αS preformed fibrils (PFF) into VCP-MSP mutation carrying mice increased phospho αS expression as compared to control mice. Cells stably expressing fluorescently tagged TDP-43 C-terminal fragment FRET pairs (TDP-43 biosensors) generate FRET when seeded with TDP-43 PFF but not monomeric TDP-43. VCP inhibition or MSP-VCP mutant expression increases TDP-43 seeding in TDP-43 biosensors. Similarly, treatment of neurons with TDP-43 PFFs generates high molecular weight insoluble phosphorylated TDP-43 after 5 days. This TDP-43 seed dependent increase in phosphorlyated TDP-43 is further augmented in MSP-VCP mutant expressing neurons.

**Conclusion:**

Using an unbiased screen, we identified the multifunctional AAA ATPase VCP as a suppressor of αS and TDP-43 aggregate seeding in cells and neurons. VCP facilitates the clearance of damaged lysosomes via lysophagy. We propose that VCP’s surveillance of permeabilized endosomes may protect against the proteopathic spread of pathogenic protein aggregates. The spread of distinct aggregate species may dictate the pleiotropic phenotypes and pathologies in VCP associated MSP.

**Supplementary Information:**

The online version contains supplementary material available at 10.1186/s13024-022-00532-0.

## Background

α-synuclein (αS) is the principal component of protein inclusions found in a family of neurodegenerative disorders known as synucleinopathies [1]. Synucleinopathies include Parkinson’s Disease, Diffuse Lewy body disease (DLB), multiple system atrophy, and REM sleep behavior disorder (RBD) [1]. αS contains an amyloid-like region making it prone to aggregate. Several lines of evidence suggest that aggregated αS can seed the fibrillization of soluble, monomeric αS [2]. This process may relate to disease pathogenesis and progression [3]. αS fibrils enter neurons via endocytosis and template new αS aggregates within the cytoplasm [4]. This leads to synapse loss, neurodegeneration, and ultimately the release of αS fibrils to adjacent cells resulting in aggregate propagation along interconnected neurons [3]. The cellular process of proteopathic seeding consists of several regulated steps. These include seed uptake, vesicular trafficking, endolysosomal escape, and templated conversion of cytosolic αS [5].

One route of seed uptake is endocytosis. αS seeds can enter cells and neurons via the endocytic system. These seeds then penetrate the endolysosomal membrane facilitating their escape into the cytoplasm [5]. Pharmacologically blocking cellular uptake or increasing endolysosomal membrane damage can decrease and increase seeding efficiency, respectively [5]. Previous studies have identified genetic modifiers of αS toxicity associated with its intracellular expression in yeast or *C. elegans* [6]. However, genetic modifiers of proteopathic seeding have not been explored.

VCP (also called p97, or cdc48 in yeast) is a ubiquitin-directed AAA-ATPase implicated in multiple forms of neurodegeneration [7]. Dominantly inherited mutations in VCP cause multisystem proteinopathy (MSP), associated with multiple variably penetrant phenotypes that include inclusion body myopathy, frontotemporal dementia, ALS, and Parkinsonism [7]. Just as the phenotypes are variable, VCP patients develop varied aggregate pathologies that include αS, TDP-43, Tau, SQSTM1, and ubiquitin inclusions [8–11]. How VCP disease mutations lead to cellular degeneration and protein inclusions is unclear. VCP affects the trafficking and clearance of polyglutamine aggregates in vitro [12]. VCP is also necessary for both autophagic and proteasomal degradation of ubiquitylated proteins, including TDP-43 and ER-associated proteins via ERAD [7]. VCP has more recently been proposed to behave as a protein disaggregase specifically acting upon pathologic tau aggregates [11]. By binding to distinct adaptors, VCP alters its functionality, allowing it to participate in its many other functions such as ERAD, vesicular trafficking, DNA repair, and cell cycle regulation [7].

VCP disease mutations alter its association with distinct adaptors [13, 14]. Specifically, VCP disease mutations have reduced binding to UBXD1 and increased interactions with Ufd1/Npl4 creating both a loss and gain of function with regard to UBXD1 and Ufd1 dependent processes [13–16]. Notably, a VCP-UBXD1 dependent complex is recruited to damaged endolysosomes [15]. This complex recruits the deubiquitinase YOD1, which cleaves K48 linked ubiquitin chains from the lysosomal membrane facilitating lysophagic degradation [15]. VCP inhibition, loss of UBXD1, or VCP disease mutant expression lead to a delay in the clearance of damaged late endosomes resulting in the accumulation of galectin-3 positive puncta in both VCP mouse models and patient tissue [15, 17].

MSP patients are pathologically characterized as a TDP-43 proteinopathy [18]. MSP patient tissue accumulates aggregated and insoluble TDP-43 in affected muscle and CNS tissue [18]. 90% of MSP patients have myopathy that precedes dementia by ~ 10 years [19]. Whether TDP-43 aggregate pathology spreads from muscle to motor neuron and ultimately the cortex is unknown. TDP-43 contains an intrinsically disordered or prion-like domain that facilitates its templated aggregate conversion [20]. Like αS, TDP-43 aggregates can serve as proteopathic seeds that propagate in cell and mouse models [21, 22].

Functional genomic screens are a powerful tool to identify proteins participating in distinct cellular pathways. In this study, we utilized an αS seeding FRET biosensor to screen a CRISPR knockout library. This approach identified multiple suppressors of αS seeding, of which the AAA ATPase, VCP, was further explored both in vitro and in vivo.

## Methods and materials

### αS FRET seeding assay

Generally, HEK 293T αS-CFP/YFP is plated in a black-bottomed 96-well plate with the density of 80 k/well in DMEM media with 10% FBS and Penicillin-Streptomycin. Three control cell lines – no-transfected HEK 293T cells, αS-CFP, and αS-YFP transduced cells are cultured in the same condition. αS PFF is sonicated and prepared with OPTI-MEM and 1 μL Lipofectamine 2000 (Invitrogen) for each well and added dropwise to the cell after 48 h. The cells are harvested after 24 h for flow cytometry, the same as reported. Briefly, the cells are detached by 0.05% trypsin-EDTA (Gibco), centrifuged, then fixed with 2% PFA for 15 min, and finally resuspended in MACSima Running Buffer. The samples are analyzed by MACSQuant® VYB. FRET signal is excited by 405 nm lasers and detected by 525/50 band pass filter. At the same time, the CFP and YFP are excited by 405 nm and 488 nm lasers and filtered by 450/50 nm and 525/50 nm, respectively. The data is analyzed with FlowJ v10 software. Each FRET signal is calculated as the percentage of FRET-positive cell timing Median FRET fluorescence intensity and then normalized to its vehicle control.

VCP mcherry vectors are a gift from Hemmo Meyer’s lab. The mutations were confirmed by Sanger sequencing (GENEWIZ) with VCP plasmid primers described before. 250 ng of the plasmid is transfected with OPTIMEM and 0.5 μL Fugene 6 (Promega) in each well 24 h after plating. The cells were then treated the same way as described above. The mcherry signal is excited by 561 nm laser and filtered via 615/20 nm, and the FRET signal is analyzed separately for mcherry positive and negative cells.

Knockdown is achieved by reverse transfection of Human SMARTPOOL siRNA from Dharmacon or Thermo Silencer Select siRNA. 6 pmol siRNA is prepared in OPTI-MEM and 0.3 μL Lipofectamine™ RNAiMAX (Invitrogen) according to its protocol and added to each well in a 96-well plate. Then 80 k suspended αS-CFP/YFP cell is plated in each well already with siRNA droplet. The αS PFF is treated 48 h after plating as described above. The concentration and duration of the drug treatments were summarized in supplemental Table 1.

### TDP-43 FRET seeding assay

TDP-43 biosensor plasmids were designed to express the glycine-rich aggregation-prone region of TDP-43 from amino acids 262 to 414. Gene expression was driven by a CMV promoter in a lentiviral FM5 plasmid (1). At the N-terminus, an alanine codon (GCG) was added to enhance expression, and the C-terminus was fused to a flexible 12-amino acid linker (GGTTCTGCTGGCTCCGCTGCTGGATCCGGCGAATTC) with mClover3 or mRuby3. Lentivirus was generated as previously described (1) and transduced to HEK293T cells to stably express both TDP-43 aa262-414-mClover3 and TDP-43 aa262-414 mRuby3. High-expressing monoclonal cell lines were sorted and tested for responsiveness to TDP-43 aggregates from TDP-43 peptides and brain homogenates from human cases with TDP-43 pathology.

TDP-43 sequence:

ATGGCGAAGCACAATAGCAATAGACAGTTAGAAAGAAGTGGAAGATTTGGTGGTAATCCAGGTGGCTTTGGGAATCAGGGTGGATTTGGTAATAGCAGAGGGGGTGGAGCTGGTTTGGGAAACAATCAAGGTAGTAATATGGGTGGTGGGATGAACTTTGGTGCGTTCAGCATTAATCCAGCCATGATGGCTGCCGCCCAGGCAGCACTACAGAGCAGTTGGGGTATGATGGGCATGTTAGCCAGCCAGCAGAACCAGTCAGGCCCATCGGGTAATAACCAAAACCAAGGCAACATGCAGAGGGAGCCAAACCAGGCCTTCGGTTCTGGAAATAACTCTTATAGTGGCTCTAATTCTGGTGCAGCAATTGGTTGGGGATCAGCATCCAATGCAGGGTCGGGCAGTGGTTTTAATGGAGGCTTTGGCTCAAGCATGGATTCTAAGTCTTCTGGCTGGGGAATG.

Generally, HEK 293T TDP-43-Ruby/Clover is plated in a black-bottomed 96-well plate with the density of 80 k/well in DMEM media with 10% FBS and Penicillin-Streptomycin. Three control cell lines – no-transfected HEK 293T cells, TDP-43 Ruby, and TDP-43 Clover transduced cells are cultured in the same condition. TDP-43 PFF is sonicated and prepared with OPTI-MEM and 1 μL Lipofectamine 20,000 (Invitrogen) for each well and added dropwise to the cell after 24 h. The cells are harvested after 48 h for flow cytometry, the same as reported. Briefly, the cells are detached by 0.05% trypsin-EDTA (Gibco), centrifuged, then fixed with 2% PFA for 15 min at dark, and finally resuspended in MACSima Running Buffer. The samples are analyzed by MACSQuant® VYB. FRET signal is excited by 488 nm lasers and detected by 614/50 band pass filter. At the same time, the Clover and Ruby are excited by 488 nm and 561 nm lasers and filtered by 525/50 nm and 615/20 nm, respectively. The data is analyzed with FlowJ v10 software. Each FRET signal is calculated as a percentage of FRET-positive cell timing Median FRET fluorescence intensity and then normalized to its vehicle control.

### Genome-wide CRISPR-Cas9 screens on αS biosensor line

αS-CFP/YFP HEK293T cells were first transduced with WT Cas9-blast. Single clones were sorted and cultured. The new cas9 αS CFP/YFP line maintained both αS-CFP and αS-YFP and was capable of seeding. Cas9 functions were validated by a synthetic gRNA and obtained with 99% NHEJ activity. About 50 million HEK293T syn CFP/YFP Cas9-blast cells were plated and then infected with pooled lentivirus with Brunello gRNA library (Addgene #73178-LV) with 8μg/ml polybrene (MOI = 0.3) the next day. After 24 h, cells would undergo 1μg/ml puromycin selection. Selected cells were replated at a density of 6.4*10^5 cell/ml after 96 h of selection and replaced with fresh puromycin. 2 days later, harvest 1/5 of the cell (~ 20 million) (untreated group, for library representation) and seeded the rest with 10 nM αS -PFF. The seeded cells were collected the same as normal αS FRET assay as described above after 24 h and sorted by Sony SY3200 cell sorter. DNA extraction was performed via QIAamp DNA Blood Midi on FRET positive and negative cells, as well as unsorted cells, which were separately amplified by PCR and deep sequenced by Illumina NovaSeq. The FRET positive and negative groups were compared with the untreated total population group separately via Megack RRA. For pathway enrichment, 154 hits were input to g:profiler and plot via Cytoscape as described before.

### αS and TDP-43 fibril preparation

αS PFF and monomer are generated as described before [23, 24]. Briefly, purified human recombinant WT αS monomer (2 mg/ml) was incubated in 20 mM Tris-HCl, pH 8.0, 100 mM NaCl for 72 h at 37 °C with shaking at 1000 rpm in an Eppendorf Thermomixer. To determine the concentration of fibrils, the fibril reaction mix was centrifuged at 18,000×g for 15 min to separate fibrils from the monomer. The concentration of αS monomer in the supernatant was determined in a BCA protein assay according to the manufacturer’s instructions, using the bovine serum albumin (BSA) standard curve. The measured decrease in αS monomer concentration was used to determine the concentration of fibrils in the 72 h fibril reaction mixture. To isolate preformed fibrils (PFF) from the monomer, centrifuge the αS mix at 18,000×g for 15 min to separate fibrils from the monomer. Resuspend fibril pellet in the buffer containing 20 mM Tris-HCl, 100 mM NaCl, pH 8.0. αS PFF was always freshly sonicated right before seeding.

Fluorescently labeled fibrils of αS were generated as previously described [25]. αS (1 mg/mL) was dissolved in 100 mM NaHCO_3_, sonicated for 15 min, and spun through a 50 kD filter (Amicon UFC5050) at 16,100×g for 15 min. Alexa Fluor 647 NHS Ester (Thermo Fisher A20006) was dissolved in DMSO to 10 mg/ml. Dye solution (molar ratio of dye: αS = 2.1:1) was pipetted into monomerized αS during stirring, and the mixture was stirred on bench for ~ 1 h. The mixture was then loaded onto a size exclusion column (Superdex 75 10/300 GL) and eluted with 5 mM NaOH. The peak containing monomeric, labeled αS was collected, aliquoted, and kept frozen until use. For aggregation assays, αS was dissolved in 10 mM NaOH at 1 mg/mL. αS-647 was added at a 5% labeling ratio. Then the solution was sonicated for 20 min, filtered through a 100 kD membrane filter (Amicon Ultra, 540,655) at 16,100×g for 15 min at 4 °C. The protein and dye concentrations were measured by absorption at 280 nm, and 647 nm, respectively, and the labeling ratio was determined to be 4.9%. To prepare labeled αS fibrils, monomer solution (5% α-syn-AF647) and solutions were incubated in 100 mM NaP, pH 7.4, 10 mM NaCl for 120 h aggregated in a non-binding 96-well plate (Corning, #3651) at a concentration of 30 μM with intermittent shaking in aggregation buffer (100 mM NaP, pH 7.4, 10 mM NaCl). A 2 mm diameter glass bead was added to each well to accelerate the aggregation through stirring. The plate was kept at 37 °C and agitated by orbital shaking once every 1 min for 5 s.

Recombinant TDP-43 (rTDP-43) was generated in *Escherichia coli* and purified as previously described [21]. Briefly, rTDP-43 was bound to nickel–nitrilotriacetic acid–agarose and washed with wash buffer 1 (50 mM Tris, pH 8.0, 500 mM NaCl, 10% glycerol, 10% sucrose, 1 mM TCEP), washed with wash buffer 2 (50 mM Tris, pH 8.0, 500 mM NaCl, 10% glycerol, 10% sucrose, 50 mM Ultrol Grade imidazole, pH 8.0, 1 mM TCEP), and finally eluted (50 mM Tris, pH 8.0, 500 mM NaCl, 10% glycerol, 10% sucrose, 300 mM Ultrol Grade imidazole, pH 8.0, 1 mM TCEP). Then, rTDP-43 was ultracentrifuged in a Beckman Coulter Optima MAX-XP Ultracentrifuge at 40,000 rpm for 30 min at 4 °C to remove any pre-existing aggregates. Soluble protein was diluted to 4uM in the reaction buffer (50 mM Tris, pH 8.0, 250 mM NaCl, 5% glycerol, 5% sucrose, 150 mM Ultrol Grade imidazole, pH 8.0, 0.5 mM TCEP). rTDP-43 aggregation was started by shaking at 1000 rpm at 22 °C for 30 min with an Eppendorf ThermoMixer C. Samples were incubated at 22 °C and collected after one to 10 days. Full-length TDP-43 recombinant protein is produced as previously described [21]. To obtain the TDP-43 monomer, TDP-43 protein was ultracentrifuge 40,000 g 30 mins at 4 °C. The supernatant was collected and freshly used.

### Primary neuronal culture

WT hippocampal neurons were obtained from E17-18 CD-1 mice (Charles River). Hippocampi are dissected in calcium- and magnesium-free Hanks’ Balanced Salt solution (HBSS) and dissociated by 0.05% Trypsin-EDTA at 37 °C for 5-10 min followed by 1% DNase I for 2 mins [26]. The cells are then resuspended with plating media to the concentration of 125 k/ml and plated on Poly-D-lysine coated plates or coverslips. The media is changed to neurobasal media (neurobasal plus + B27 + 5 mM L-Glutamine) after 2-4 h. The culture was treated with 1 mM Ara-C to inhibit the growth of glia at DIV3. The αS PFF is sonicated and added directly to the cell at DIV10.

VCP and UBXD1 shRNA is delivered via lentivirus. Lentivirus were added to the neurons at DIV5 with MOI ≥ 1. For LLoMe or VCP inhibitors experiments, drug or vehicle control was added simultaneously with αS PFF or monomer (1μg/mL) for 4 h. Then the media is fully exchanged to the conditioned neurobasal media without drug and αS PFF or monomer. The neurons were harvested at DIV15.

R155H/WT neurons were cultured from embryos from R155H/WT intercross. The hippocampus from each embryo was dissected and cultured separately and then plated at the same density. The genotypes were examined by PCR (Transnetyx) (Forward Primer: CCTCTAATTGCACTTGTATTGCTTTGT; Reverse Primer: CTGGGATCTGTCTCTACAACTTTGA).

### Immunohistochemistry

Cells were fixed in 4% PFA for 10 mins and permeabilized with 0.1% Triton X-100 in PBS for 10 mins. Then the cells were blocked with 2% BSA in PBS at RT for 1 h. Cells were stained with primary antibody at 4 °C overnight, followed by three washes with PBS. Cells were then incubated with the Alexa 488, 555 or 647 tagged secondary antibody in 1:500 dilution for 1 h at RT. The nucleus was stained with DAPI (1:1000) for 10 min at RT. After three wash with PBS, the cells are mounted by Mowiol. Pictures were taken by Nikon Eclipse 80i fluorescence microscope and processed via ImageJ. The antibody is listed in supplement Table 2.

The FRET images were taken under Olympus FluoView1200 confocal microscope. The laser and band filters were set as listed:

**Table:** 

Channel	Excitation wavelength (nm)	Band filter (nm)
αS Biosensor line		
CFP	440	425-475
YFP	488	500-550
FRET	440	500-550
TDP-43 Biosensor line		
Clover	488	500-550
Ruby	559	605-625
FRET	488	605-625

### Immunoblot

Mouse cortex or cells are lysed in RIPA buffer with protease inhibitor cocktails (PMSF and PIC) followed by two 30 s on and 30 off sonication cycles at 50% power. The protein concentration is normalized by the BCA assay. Samples were loaded into 10 to 15% gel and transferred into nitrocellulose or PVDF membrane. The membranes were blocked by 5% milk in PBS-0.2% Tween20 and incubated with the primary antibody in blocking solution overnight at 4 °C degree. The membrane was then washed three times with PBS-0.2% Tween20 and incubated with secondary goat anti- rabbit or mouse HRP antibody (1:5000) for 1 h. Blot was rinsed three times with PBS-0.2% Tween20 and probed by a fresh mixture of ECL reagents at dark and then exposed by SYNGENE.

To fractionate the insoluble portion of αS, we performed sequential extraction as described [27]. Briefly, neurons were first dissolved in TBS-1%Tx-100, and sonicated for ten cycles of the 30s on, 30s off with 50% power. The lysate would incubate on ice for 30 mins. 1/10 of the lysate was saved as total protein, while the remains were ultracentrifuged 100,000 g 4 °C for 30 mins. The supernatant was collected as Tx- 100 soluble fraction. The pellet was washed with TBS-1%Tx-100, sonicated, and ultracentrifuged. Ultimately, the pellet was resuspended with TBS-2%SDS and sonicated for 15 cycles of 30s on, 30s off. Soluble and insoluble fraction were run by western blot as usual. The loading amount was determined by the concentration of total protein measured by BCA.

TPD-43 soluble and insoluble extraction is done as our previous method. Briefly, neurons from one 35 mm dish were first lysed with RIPA buffer with protease inhibitor cocktails (RIPA buffer) on ice. The lysate was then sonicated with QSONICA sonicator for ten cycles of 30s on, 30s off with 50% power. 1/10 of the lysate was saved as total protein. The rest was ultracentrifuged at 100,000 g 4 °C for 30 mins. The supernatant was kept as RIPA soluble fraction. The pellet was then washed with RIPA buffer once, resonicated, and ultracentrifuged with the same condition. The pellet was finally resuspended with the same amount of UREA buffer (30 mm Tris, pH 8.8, 7 m urea, 2 m thiourea, and 4% CHAPS) as an insoluble fraction. Soluble and insoluble fraction were run by western blot as normal. The loading amount was determined by the concentration of total protein measured by BCA.

### Animals

C57BL/6 (stock No.: 000664) and VCP^R155H/WT^ (B6;129S-Vcptm1Itl/J, Stock No: 021968) were purchased from Jackson Laboratory. To obtain VCP cKO (VCPR155C/FL; Rosa26-CRE^ERT2^), we first crossed VCP ^flox;flox^ with Rosa26-CRE^ERT2^ (B6.129-Gt (ROSA)26Sortm1(cre/ERT2)Tyj/J, Stock No: 008463) to get VCP ^flox;wt;^ Rosa26-CRE^ERT2^. Then we bred those mice with VCP^R155C/WT^ reported previously for VCP cKO [28]. All mice utilized in the study and breeding were on a C57BL/6 background. Both male and female mice were used in this study. Animal procedures were performed in accordance with protocols approved by the Animal Studies Committee at Washington University School of Medicine.

### Intrastriatal injection and mouse brain harvest

Both mice are Intraperitoneal injected with 75 mg tamoxifen/kg body weight at 10 weeks of age and wait 1 month for gene knockdown. The R155C mutation allele and the sufficiency of VCP flop knockdown were confirmed by PCR. Intrastriatial injection is performed as described. αS PFF is prepared as described above and diluted in sterile PBS. αS PFF is sonicated 10 mins before injection. The mouse is anesthetized and injected at the dorsal striatum (Bregma = 0.2 mm, midline = 2.0 mm, depth = − 3.2 mm) of the left hemisphere. The same amount of PBS is used as vehicle control. The recovery of mice is monitored in the following week and sacrificed after 90 days. The mouse was first anesthetized in the Isofluorane chamber and perfused with PBS containing heparin. The whole brains were removed from the skull and fixed in 4% PFA overnight at 4 °C degree and cut coronally into 40 μm sections and stored in cryoprotectant solution at 4 °C degree for staining. Sections were first rinsed three times with TBS and then blocked with blocking solution for 30 min (5% normal goat serum with 0.1% Triton X-100 in TBS). Sections were stained with the primary antibody in TBS-0.1% Triton X-100 plus 2% normal goat serum at 4 °C overnight, followed by three washes with TBS. Sections were then incubated with the Alexa 488,555 tagged secondary antibody in 1:1000 dilution for 2 h at RT. The nucleus was stained with DAPI (1:1000) for 20 min at RT. After three wash with TBS, the sections were mounted on the glass slides. True black (cat: NC1125051) was incubated with the sections for 5 min to quench the auto-fluorescence. Finally, slides were coverslipped using Prolong Gold mounting medium. Pictures were taken by a Hamamatsu NanoZoomer or Nikon Eclipse 80i fluorescence microscope. The images were processed, and fluorescence intensity was calculated via ImageJ.

### Antibodies

All antibodies used in this study are listed in supplemental Table 2.

### Statistical analysis

The data (except CRISPR screening) is analyzed by GraphPad Prism 9. Statistical tests included unpaired t-test, one-way ANOVA, multiple t-test, linear interpolation (95% confidence), and two-way ANOVA. Data were displayed as mean ± SEM. Two-stage step-up methods of Benjamini Krieger and Yekutieli, Dunnett, Sidak correction were used to minimize false alarm from multiple comparisons.

## Results

### Genome-wide CRISPR knockout screen identifies genes protective against αS seeding

**Fig. 1:**
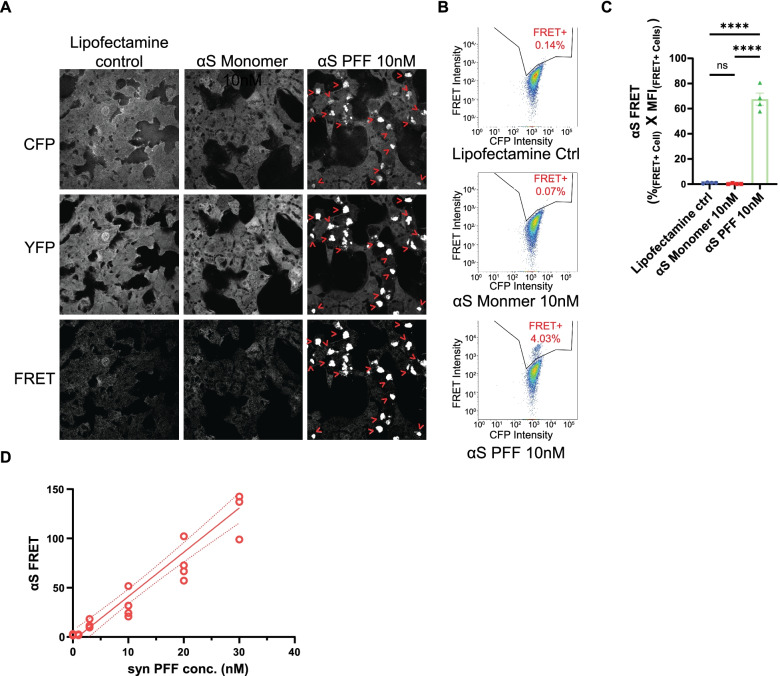
αS seeding can be sensitively detected by FRET in αS biosensor line. **A** Representative FRET confocal microscopy images of αS biosensor line (αS CFP/YFP) treated with Lipofectamine (left, ctrl), 10nM αS monomer (center) and αS PFF (right) after 24 hours. Scale bar= 10μm. **B** Flow cytometry tracing of FRET signal from αS biosensors. FRET+ gate (CFP vs FRET) was drawn from empty Lipofectamine treated cells with no aggregation. **C** Quantitation of integrated FRET signal from FRET-flow cytometry is calculated as % FRET+ cell * Median (FRET+ Intensity). **** *p* < 0.0001, n.s., no significance by one-way ANOVA. Error bars are ±S.E.M. **D** Quantitation of cells treated with increasing concentrations of αS PFF for 24 hours. Cells are harvested after 24 hours and analyzed the same as 1C. Each dot represents an independent experiment

To identify genes that regulate αS seeding, we utilized a previously described HEK293 αS CFP/YFP biosensor cell line (αS biosensor) (Fig. 1) [4]. These cells stably co-express two αS constructs. One tagged with CFP (donor) and another with YFP (acceptor). We exogenously applied human WT αS PFF or monomer with Lipofectamine for 24 h to the αS biosensor line. Only αS PFF treated, but not monomer or empty Lipofectamine treated cells, induce aggregation of soluble intracellular αS, as shown by CFP and YFP positive aggregates. This PFF-dependent process is referred to as a seeding activity. CFP/YFP positive aggregates can be visualized by FRET under confocal microscopy (excitation = 440 nm, emission = 500-550 nm) (Fig. 1A, lower panel). Quantitation of the percent of FRET+ cells and FRET intensity can be detected using flow cytometry (Fig. 1B) [29]. The FRET efficiency, measured as %_(FRET + cells)_ * Median Fluorescent Intensity (MFI) _(FRET + cells)_, was significantly higher in αS PFF treated group as compared with Lipofectamine control and αS monomer controls (mean = 67.55 vs. 0.96 vs. 0.30) (Fig. 1C). In addition, αS PFF induced FRET efficiency is sensitive and quantitative in a concentration-dependent manner (Fig. 1D).

**Fig. 2:**
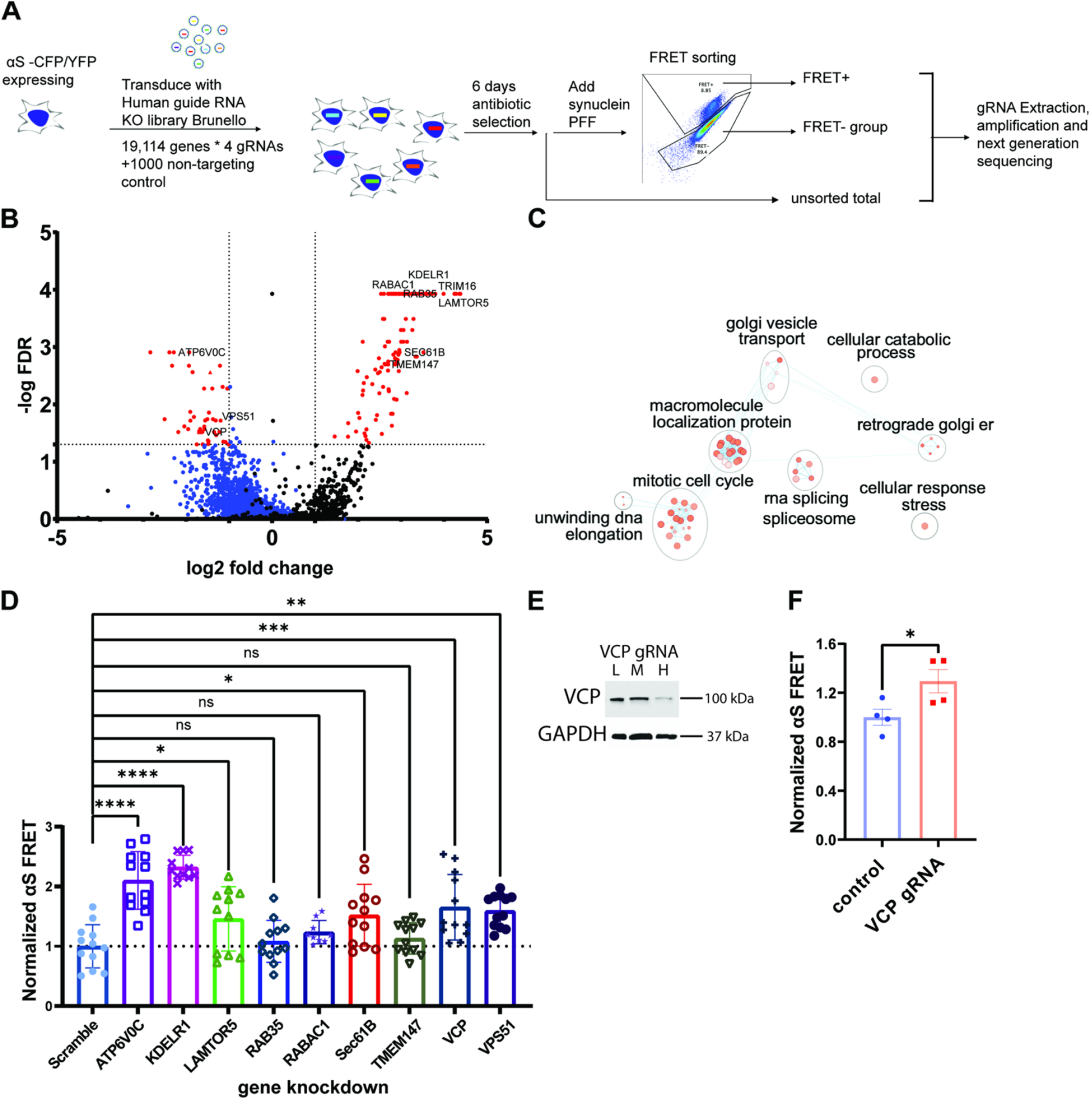
Genome-wide CRISPR/Cas9 screen identifies genes protective to αS seeding. **A** αS biosensor line stably expressing spCAS9 was transduced with sgRNA lentiviral library (Brunello). Following antibiotic selection, biosensors were seeded with αS PFF (10nM) and flow sorted 24 hours later. Genomic DNA from positive and negative groups as well as unsorted total population were collected and decoded by NGS. **B** Volcano plot of genes identified in the screen. Colored in blue are all the genes plotted from FRET- group, while colored in black are those plotted from FRET+. Red dots are protective hits from both groups (5% FDR), specifically genes with a fold change <0.5 that were underrepresented in FRET- cells and genes >2 that were overrepresented in the FRET+ cells. **C** Pathway analysis of 154 protective genes via g:profiler. The enriched pathway is visualized by cytoscape. **D** Normalized FRET from αS biosensors following siRNA knockdown of 9 genes identified in the screen and αS PFF treatment. (n ≥9 repeats ; *** *p* < 0.001, ** *p* < 0.01 and * *p* < 0.05 by one-way ANOVA. error bars are ± S.E.M.). **E** Immunoblot with anti-VCP and anti-GAPDH of cell lysates from αS biosensors/spCas9 cells treated with a VCP gRNAs at low (L) medium (M) and high (H) concentration and harvest at 6 days after transduction demonstrates VCP at 16.5% non-treated controls in the high concentration treatment. **F** Normalized FRET of αS biosensors/spCas9 cells treated with a VCP gRNA or scrambled control gRNA following 24-hour application of αS PFF. (*p* = 0.0439 by a two-tailed student’s t-test). *n* = 4 biologically independent FRET assay. Data presents as mean ± S.E.M

To perform our screen, we clonally expressed spCAS9 in the αS biosensor line and then infected with a pooled Brunello gRNA library covering 19,114 different genes/ 4 gRNA each and 1000 non-targeting controls at a low MOI (< 0.3) for 7 days with puromycin selection (Fig. 2A). The pooled knockdown αS- spCAS9 biosensor maintained αS seeding capacity in a concentration-dependent manner (Fig. S1). These biosensors were treated with 10 nM αS PFF and flow-sorted into FRET positive and FRET negative groups. DNA was isolated from FRET positive, FRET negative, and the unseeded total cell population. Then deep sequencing was performed to identify the guide RNAs represented in each group (Fig. 2A). This sequence data was analyzed via the MegaCK algorithm in both the FRET positive and negative populations compared with untreated control. 111 genes were enriched in the FRET positive population as compared to the total population, and 43 genes were underrepresented in the FRET negative group versus total population (FDR < 0.05 and fold change > 2 or < 0.5) (Fig. 2B). These 154 genes (red dots) were considered “protective” or suppressors of αS seeding in the biosensor line (Fig. 2B-C; Table S3).

The screen identified genes and pathways previously identified in other αS screens for αS toxicity. Notably, we identified 15 genes associated with ER-Golgi-endosome trafficking. These included VPS51 and VPS52, which are components of the Golgi-associated retrograde protein (GARP) complex. The GARP complex interacts with PD-associated protein, LRRK2, and deletion of either VPS51 or VPS52 homologs in yeast increases αS accumulation and toxicity [30]. Other modifiers not previously identified in screens include ATP6V0B, ATP6V0C, and ATP6V1A that encode subunits of Vacuolar-type ATPase (V-ATPase). ATP6V0B KD inhibits autophagic degradation and increases αS aggregation [31] and is downregulated in patients with αS inclusions [32]. Pathway analysis identified an enrichment in genes associated with the cellular stress response, such as VCP, SEC61B, and KDELR1. Notably, the ER stress response is upregulated in PD brains and correlates with αS toxicity in multiple model systems [33].

We validated nine candidate suppressors from both FRET positive and negative selections (ATP6V0C, KDELR1, LAMTOR5, RAB35, RABAC1, SEC61B, TMEM147, VCP, and VPS51) using siRNA knockdown in αS biosensors. Following 48 h of siRNA treatment, αS PFF (10 nM) was added with Lipofectamine, and FRET efficiency was measured 24 h later. Six candidate suppressors, when knocked down, increased FRET efficiency and included ATP6V0C, VPS51, KDELR1, SEC61B, LAMTOR5, and VCP (Fig. 2D). To further confirm our findings with VCP, we generated a lentiviral vector expressing a VCP specific or control gRNA, infected spCAS9 αS biosensors for 7 days and treated with αS PFF. Similar to that seen with VCP siRNA, FRET efficiency was increased in VCP CRISPR KO αS biosensors (Fig. 2E-F).

### VCP inhibition increases α-synuclein seeding efficiency

**Fig. 3:**
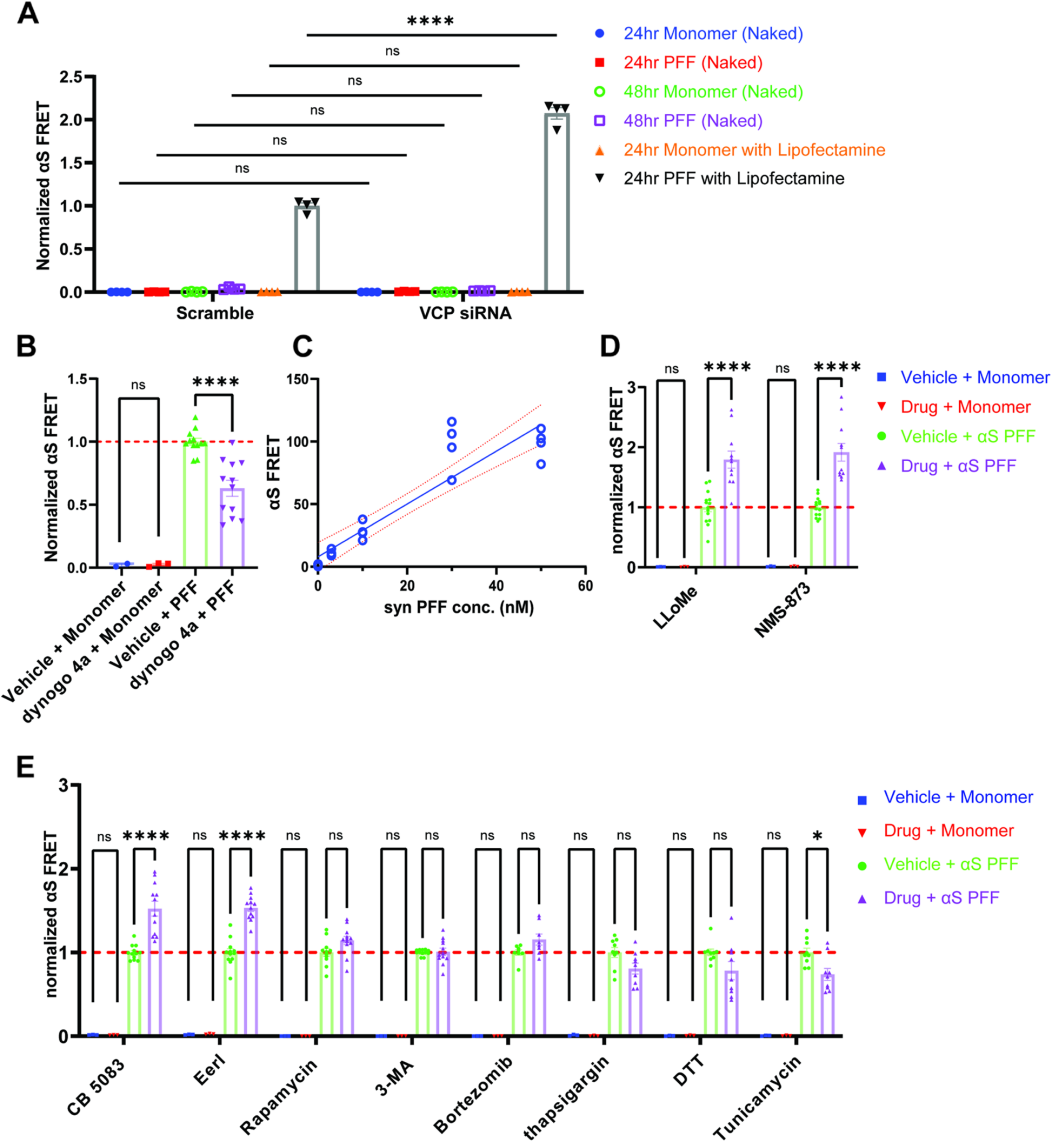
VCP inhibition enhances αS seeding. **A** αS biosensors were treated with scrambled control siRNA or VCP siRNA for 48 hours prior the application of αS monomer or PFF (10nM) with or without Lipofectamineand measured for FRET efficiency at 24 and/or 48 hours. *N*=4 for each groups.; n.s.= no significant, **** *p* < 0.0001 by two-way ANOVA compared with scramble siRNA in each conditions. error bars are ±S.E.M.). **B** αS biosensors were treated with Dynogo4a for one hour prior to αS PFFormonomer (10nM) treatment with Lipofectamine. n =12 repeats for PFF treated conditions. **** *p* < 0.0001; n.s. for monomer treated pairs by two-way ANOVA. **C** αS biosensors treated with αS PFF for 4 hours followed by media exchange and washout. FRET signal was measured the same as in 3A, 20 hours after washout. n.s. no significant and **** *p* < 0.0001 compared to vehicle washout at different concentrations. Each dot represents an independent experiment. **D** Normalized FRET of αS biosensors co-treated with αS monomer or PFF (30nM) with the indicated chemical compound or vehicle for four hours followed by washout and FRET measurement 20 hours later. (*n* =14 repeats for each PFF treated conditions; n.s. for all monomer treated pairs; **** *p* < 0.0001 by two-way ANOVA in some PFF treated pairs as indicated. error bars are ±S.E.M.). **E** Normalized FRET of αS biosensors co-treated with αS monomer or PFF (10nM) with the indicated chemical compound or vehicle for 24hours. FRET signal was obtained same as3A (*n* ≥8 repeats for each PFF treated conditions; n.s. for all monomer treated pairs; **** *p* < 0.0001 and * *p* < 0.05 by two-way ANOVA in some PFF treated pairs as indicated. error bars are ±S.E.M.)

As shown in Fig. 1, αS seeding as measured by FRET is not seen with the application of monomeric αS [4]. Consistent with this, VCP siRNA treated αS biosensors showed no increase in FRET signal when treated with αS monomer (10 nM) as compared with control siRNA treated (Fig. 3A). Moreover, Lipofectamine is necessary for efficient FRET in this assay (“Lipo” seeding). The application of αS PFF (10 nM) directly to the media (“naked” seeding) fails to induce robust aggregation and FRET signal after 24 and 48 h, with less than 0.2% of cells being FRET positive (data not shown) [4]. Notably, VCP KD did not increase “naked’ seeding and only augmented “Lipo” seeding when compared with scrambled control KD (Fig. 3A). To understand whether αS PFF enter through the endocytic pathway with “Lipo seeding”, we pre-treated cells with Dynogo-4a (10 μM), a dynamin I/II inhibitor, for 1 hour before αS PFF with Lipofectamine application for 24 h. Consistent with previous studies showing that “Lipo” αS PFF internalization is dynamin-dependent [34], we found a decrease in FRET with Dynogo-4a (Fig. 3B). Notably, the increase in FRET efficiency with VCP KD was Dynogo-4a inhibitible suggesting that this increase occurred within the endocytic pathway (Fig. S2). Subsequent studies using the αS biosensor line are performed using “Lipo” seeding (Fig. 3B-E; 7A).

In order to probe the role of VCP specifically at the time of endocytic entry into the cytosol, we modified our seeding protocol to a four-hour application of αS with Lipofectamine and subsequent washout of αS PFF from the media. Seeding activity is quantified via FRET-Flow after 20 h. This four-hour application was sufficient to seed αS aggregation as measured by FRET in a concentration-dependent manner (Fig. 3C). A four-hour treatment at the time of αS PFF application with the lysosomal permeabilizing agent (LLoMe, 1 μM, 4 h) significantly increased FRET (Fig. 3D). Treatment of αS biosensors with the VCP inhibitor NMS-873 [35] for 4 hours at the time of αS PFF application similarly increased seeding efficiency as measured by FRET (Fig. 3D).

We further validated the effect of VCP inhibition with two additional VCP inhibitors, CB-5083 [36] and Eeyarestatin I (Eer1) [37]. Continuous application of either inhibitor with αS PFF increased the seeding efficiency as measured by FRET (Fig. 3F). VCP inhibition has been show to effect both the autophagic and proteasomal pathways [38]. 24-h application of the proteasome inhibitor Bortezomib or autophagy modulators (100 nM Rapamycin and 1 μM 3-methyladenine (3-MA)) with αS PFF did not affect seeding efficiency (Fig. 3F). Finally, both VCP inhibition and knockdown can induce ER stress and activate the unfolded protein response (UPR) [38, 39]. Treatment of αS biosensors with the ER stress inducing agents dithiothreitol (DTT, 1 mM, 24 h), thapsigargin (0.3 μM; 24 h), and tunicamycin (2.5 μg/mL, 24 h) with αS PFF (10 nM) for 24 h had no effect or decreased FRET efficiency as compared to vehicle controls. This suggested that the effect of VCP inhibition on seeding is ER stress independent (Fig. 3E). Notably, no treatment significantly altered FRET efficiency compared with vehicle control when αS monomer was added (blue and red bars, Fig. 3E), indicating that these effects are αS PFF dependent.

**Fig. 4:**
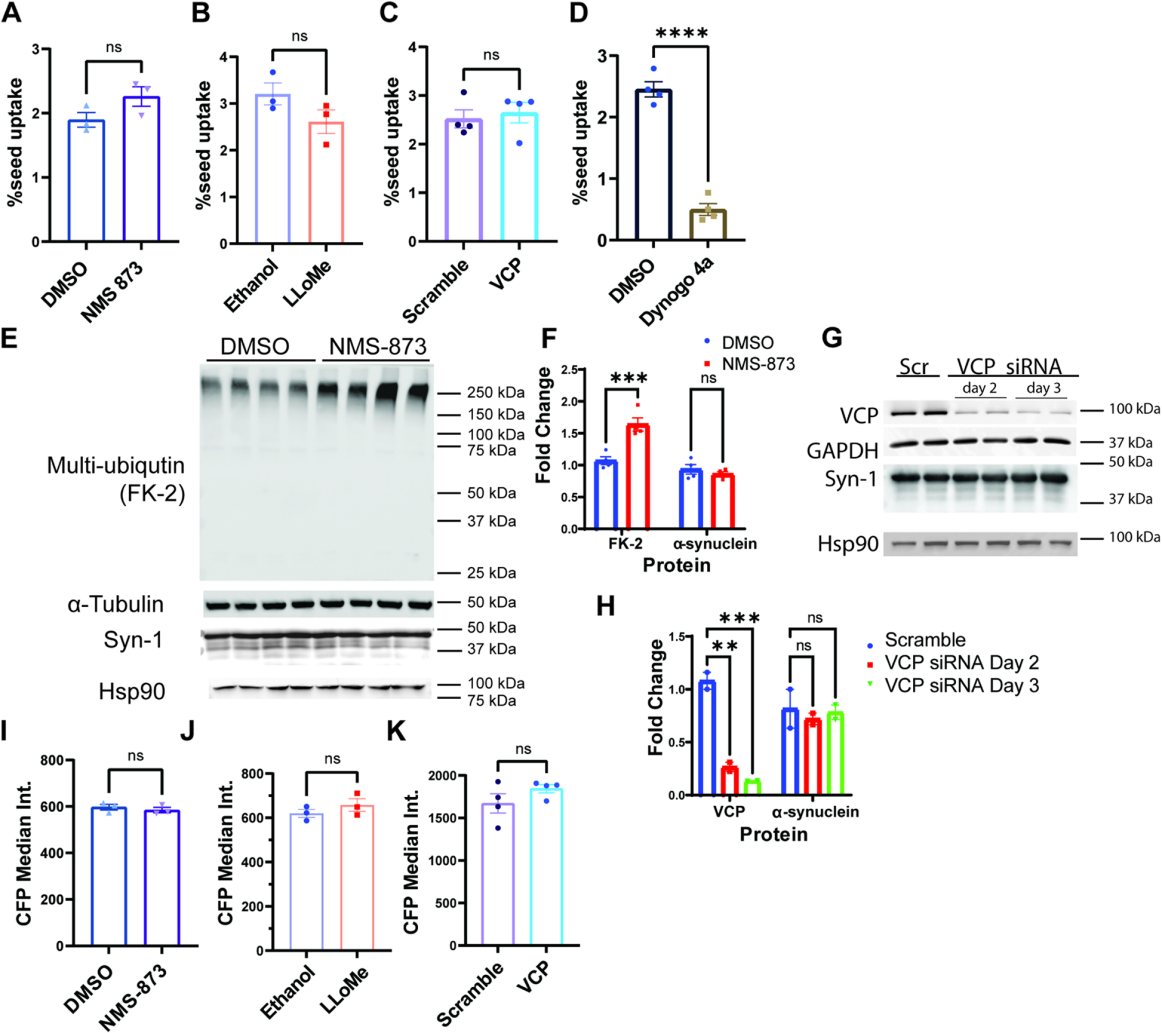
VCP inhibition does not affect seed uptake or αS protein levels. **A** Quantification of percentage of Alexa-647 positive cells (seed uptake) from αS biosensors co-treated with Alexa-647 tagged αS PFF together with vehicle or NMS-873 (1μM) for 4 hours. *n*=3 biological repeat. Data is mean ±S.E.M. n.s. no significant from student’s t test. **B** Quantification of seed uptake from αS biosensors co-treated with Alexa-647 tagged αS PFF and LLoMe or vehicle for 4 hours. *n*=3 biological repeat. Data is mean ±S.E.M. n.s. no significant from student’s t test. **C** Quantification of seed uptake from VCP or scramble siRNA treated cells with 4-hour Alexa-647 tagged αS PFF application. *n*=4 biological repeat. Data is mean ±S.E.M. n.s. no significant from student’s t test. **D** Quantification of seed uptake from αS biosensors treated with Dynogo-4a or DMSO for one hour and then Alexa-647 tagged αS PFF for four hours. *n*=4 biological repeat. Data is mean ±S.E.M. ****p < 0.0001 from student’s t test. **E** Immunoblot of anti-Ubiquitin (FK-2), and total α-synuclein (syn-1) from αS biosensors treated for four-hours with NMS-873 (5μM) or DMSO. α-tubulin and HSP90 are loading controls. **F** Quantitation of band intensities of VCP and α-synuclein in E. *** *p* < 0.001 and n.s.= no significant by two-way ANOVA. **G** Immunoblot of anti-VCP, and α-synuclein (syn-1) from αS biosensors treated with scrambled or VCP siRNA for 48 and 72 hours. GAPDH and HSP90 are loading controls. **H** Quantitation of band intensities of VCP and α-synuclein in G. ** *p* < 0.01 *** *p* < 0.001 and n.s.=no significant by two-way ANOVA. **I** Quantification of CFP fluorescent intensity of NMS-873 treated cells. Drug treatment and timeline were the same as 3D. *n*=3 biological repeat. Data is mean ±S.E.M n.s.no significant from student’s t test. **J** Quantification of CFP fluorescent intensity of LLoMe treated cells. Drug treatment and timeline were the same as 3D. *n*=3 biological repeat. Data is mean ±S.E.M. n.s. no significant from student’s t test. **K** CFP fluorescent intensity of αS biosensors treated with scrambled or VCP siRNA. *n*=4 biological repeat. Data is mean ±S.E.M

To see whether the increased seeding efficiency with VCP inhibition was due to an increase in αS PFF uptake, we employed a fluorescently conjugated αS PFF (αS-PFF 647). αS-PFF 647 retains seeding capacity in αS biosensors (Fig. S3). The dynamin I/II inhibitor Dynogo-4a significantly decreased αS PFF 647 uptake when measured at 4 h post seed application (Fig. 4A). In contrast, four-hour αS biosensor treatment with αS-PFF 647 in the presence of LLoMe or NMS-873 did not increase the amount of internalized αS PFF 647, as quantified by the percentage of Alexa 647 positive cells via flow cytometry (Fig. 4B-C). Uptake was also unchanged when comparing scrambled and VCP siRNA KD cells (Fig. 4D). In addition, the increased seeding efficiency with VCP chemical inhibition or VCP siRNA knockdown was not due to an increase in the steady-state levels of soluble αS, as determined by immunoblot against total αS and αS fluorescence intensity as measured by CFP median fluorescent intensity via flow cytometry (Fig. 4E-K). Notably, VCP siRNA KD was > 75% and a 4 hour treatment with NMS-873 was sufficient to increase the level of high molecular weight (HMW) ubiquitin conjugates consistent with VCP inhibition (Fig. 4E-H).

**Fig. 5:**
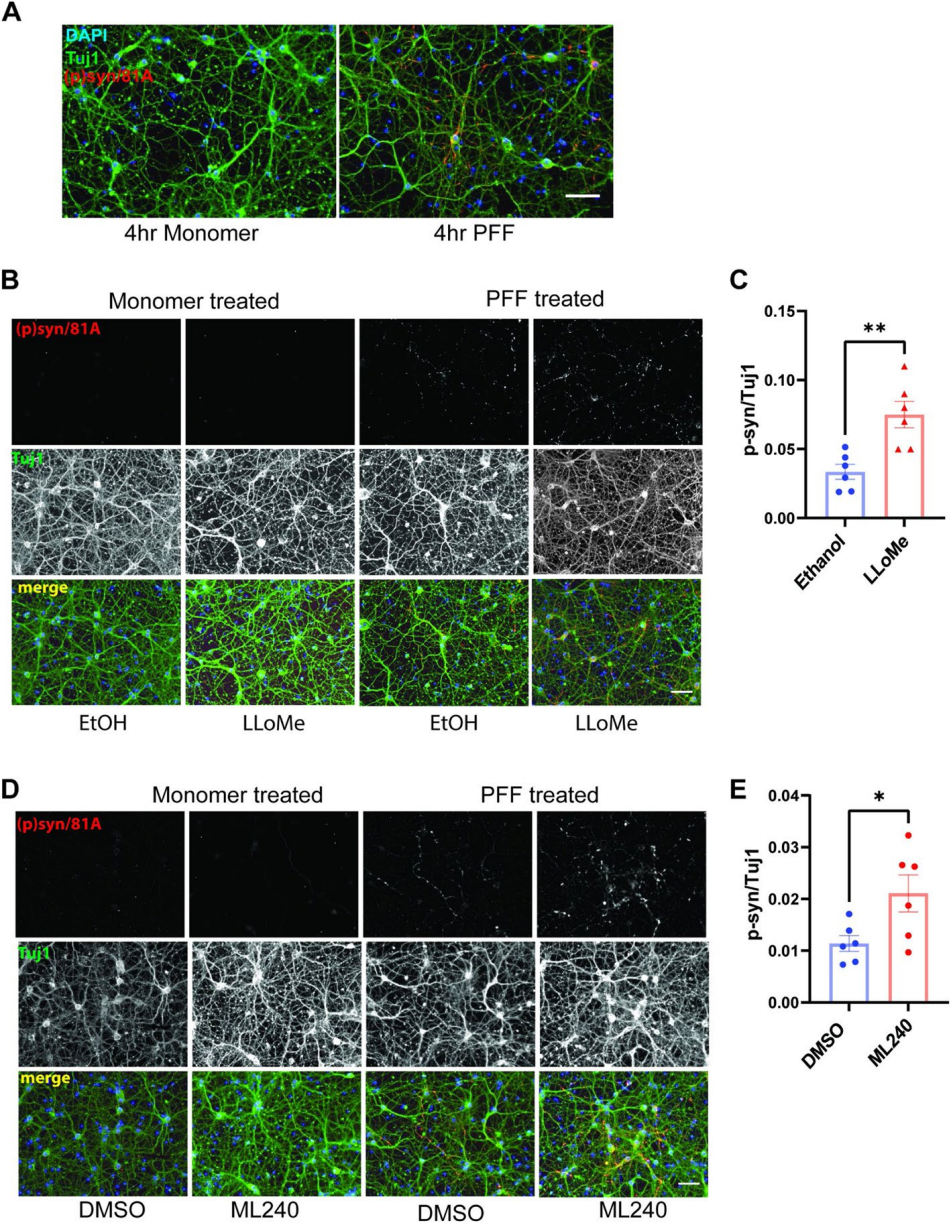
VCP inhibition enhances αS seeding in neurons. **A** Immunofluorescence for phospho-αS and Tuj1 (neurite marker) in HNs treated for 4 hours with αS PFF or monomer (1μg/ml) followed by washout and harvested 5 days later. **B** Immunofluorescence for phospho-αS and Tuj1 in HNs co-treated with αS PFF or monomer (1μg/ml) and LLoMe (1µM) or vehicle for 4 hours. Immunostaining wasperformed after 5 days. Scale bar =20μm. **C** Quantitation of phospho-αS/Tuj1 staining in 5B. Quantitation in 5B and subsequent HN studies were performed using the average intensity of multiple fields from individual coverslips. Each coverslip was treated as an independent experiment. *n*=6 for ethanol and LLoMe groups respectively. Experiments were repeated from 3 different cultures. **p< 0.01 by student’s t-test. Data are mean±S.E.M. **D** Immunofluorescence for phospho-αS and Tuj1 in HNs co-treated with αS PFF or monomer (1μg/ml) and ML240 (100nM) or DMSO control for 4 hours and harvested after 5 days. Scale bar =20μm**. E** Quantitation of phospho-αS/Tuj1 staining in 5D. *n*=6 for DMSO and ML240 group. Experiments were repeated from 3 different cultures. *p< 0.05 by student’s t-test. Error bars are ±S.E.M.)

**Fig. 6:**
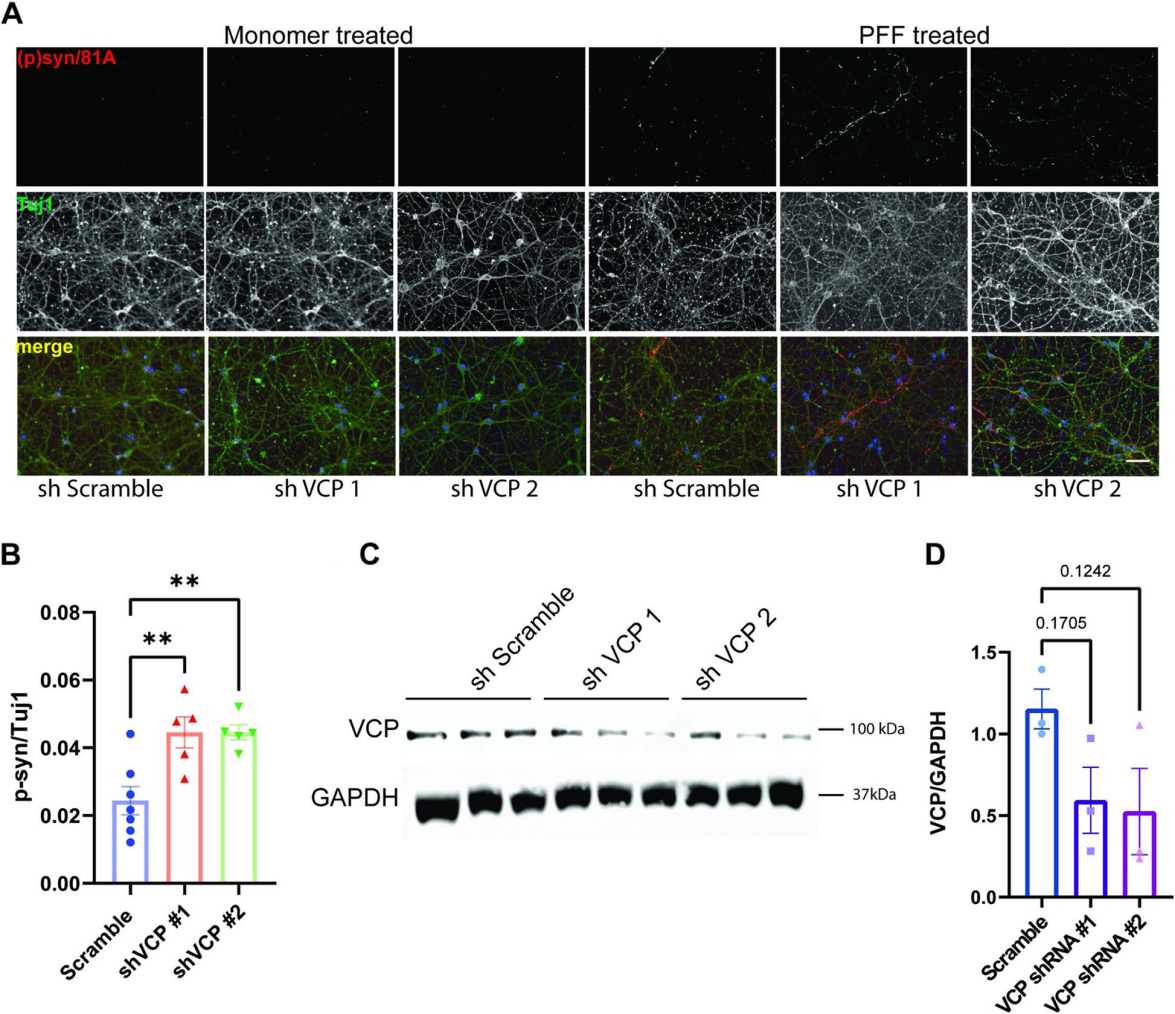
VCP knock down enhances αS seeding in neurons. **A** Immunofluorescence for phospho-αS and Tuj1 in HNs infected with lentiviral vectors expressingscrambled or one of two different shRNAs targeting VCP for 5 days and then treated with αS PFF or monomer (1μg/ml) for 5 days. Scale bar =20μm. **B** Quantitation of phospho-αS/Tuj1 staining in 3F. Experiments were repeated from 3 different cultures. N=7,5 and 5 for scramble and VCP shRNA 1 and 2 respectively. **p < 0.01 one-way ANOVA with Dunnett correction. Error bars are ± S.E.M. **C** Representative immunoblot from independent HN lysates treated with scramble or VCP shRNAs for anti-VCP and GAPDH. **D** Quantitation of band intensities of VCP over GAPDH control

To explore the role of VCP in a more relevant system of αS seeding that does not require the use of the carrier Lipofectamine, we adapted a previously described assay that adds αS PFF to primary cultured hippocampal neurons (HNs) [40]. Previous studies show that exogenously applied αS PFF can induce endogenous αS aggregates in HNs that are hyperphosphorylated and Tx-100 insoluble. To avoid continuous αS PFF application, we added αS PFF to the media of HNs for only 4 hours, followed by washout with conditioned media. A four hour application of αS PFF versus αS monomer was sufficient to generate phospho-αS after 5 days as shown by immunofluorescence using a phospho-αS (phospho Ser129/81A) antibody in HNs (Fig. 5A). This four-hour treatment allows us to manipulate the effect of VCP inhibition at the time of seed uptake. We then co-treated HNs with LLoMe (1µM) and αS PFF (1 μg/ml) for 4 h followed by washout, which further increased the amount of phospho-αS as compared with the application of αS PFF and vehicle (Ethanol) after 5 days (Fig. 5B-C). These results were αS PFF dependent since treating HNs with LLoMe and monomeric αS (1 μg/ml) failed to generate phospho-αS aggregates (Fig. 5B). We performed a similar assay and treated HNs for 4 hours with the reversible VCP inhibitor ML240 [41] (100 nM) and αS PFF (1 μg/ml). Notably, the application of ML240 for 4 hours at the time of αS PFF application increased the level of phospho-αS as compared with vehicle-treated control, while ML240-treated HNs with αS monomers showed no phospho-αS signal (Fig. 5D-E). We further validated the role of VCP with two different shRNAs against VCP. Primary HNs were treated with lentiviral shRNA (at DIV5) for 5 days prior to a five-day continuous application of αS PFF or monomer treatment (at DIV10). Both VCP shRNAs decreased VCP protein levels and demonstrated an increase in an αS PFF-dependent increase in phospho-αS staining compared with scrambled shRNA control (Fig. 6A-D). Treatment with LLoMe, ML240, or shRNA-VCP treatment did not alter cell viability as measured by MTT assay after 10 and 15 days in culture (Fig. S5).

### VCP disease mutations increase αS seeding in vivo

**Fig. 7:**
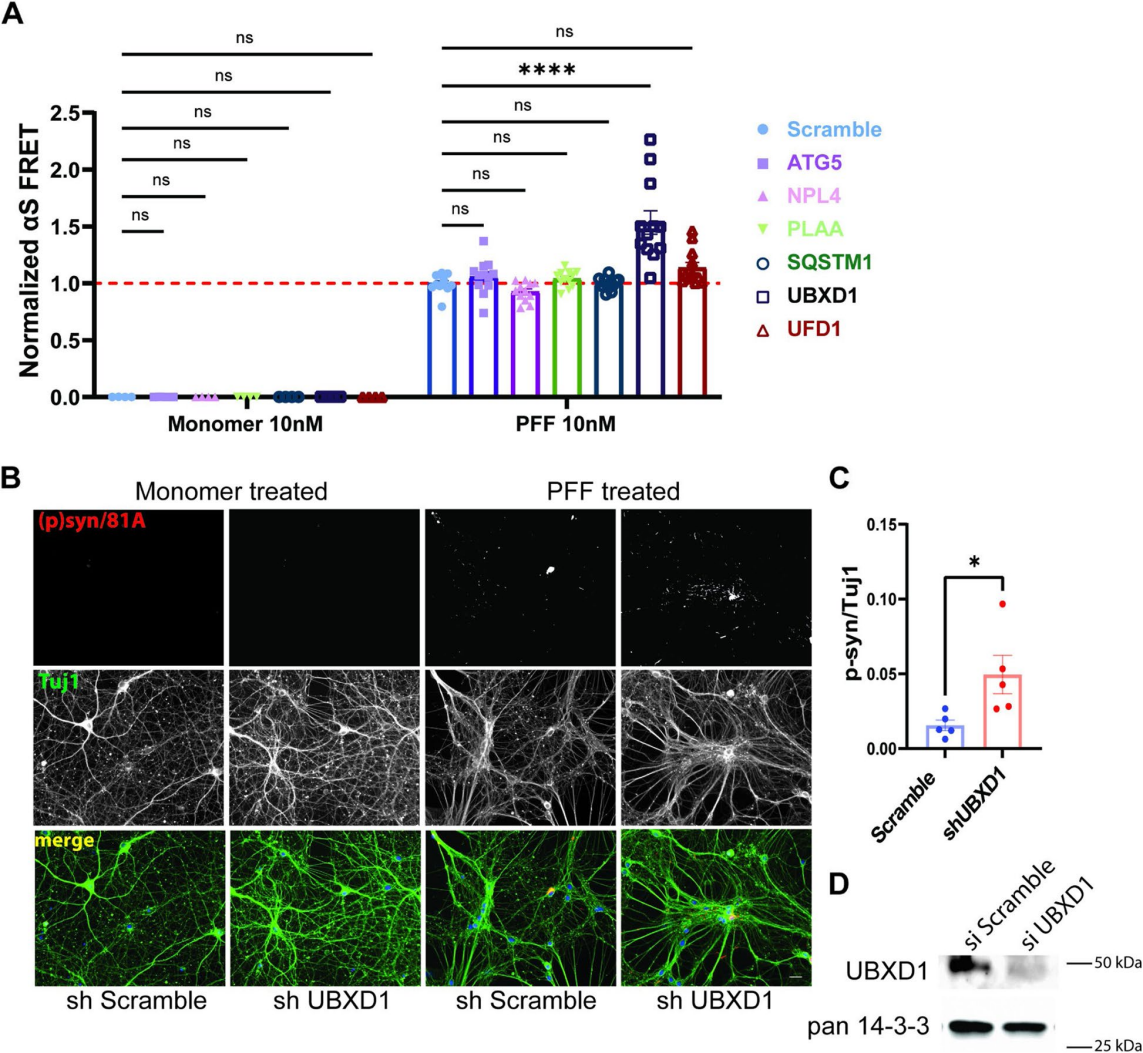
VCP cofactor UBXD1 knockdown augments αS seeding. **A** αS biosensors were treated with scrambled control siRNA or siRNAs targeting the indicated genes for 48 hours prior to the application of αS PFF or monomer (10nM) and harvested for FRET efficiency at 24 hours. *n* =4 and 12 for the monomer and PFF treated group respectively for each KD. Adjusted *p* ****p < 0.0001 for UBXD1 by two-way ANOVA with Šidák correction; n.s.=no significance. Error bars are± S.E.M. **B** Immunofluorescence for phospho-αS and Tuj1 in HNs infected with lentiviral vectors expressing scrambled or a shRNA targeting UBXD1 for 5 days and then treated with αS PFF or monomer (1μg/ml) for 5 days Scale bar =20μm. **C** Quantitation of phospho-αS/Tuj1 staining from PFF treated HNs infected with scramble or control UBXD1 shRNAs. Experiments were repeated from 3 different cultures. *N*= 5 for each groups. * *p*<0.05 by Student’s t test). **D** Representative immunoblot from HN lysates treated with scramble or UBXD1 shRNAs for anti-UBXD1 and α-tubulin

VCP disease mutations affect a subset of VCP dependent cellular processes such as endocytic trafficking, nutrient sensing, autophagosome maturation, and, more recently, lysophagy [7, 15]. This is due to an impairment in VCP mutant association with the adaptor UBXD1 [13]. We performed siRNA knockdown of VCP adaptors UFD1, NPL4, UBXD1, and PLAA and the autophagy proteins ATG5 and SQSTM1 along with a scrambled control in αS biosensors (Fig. S4A). Following 48 h of knockdown, αS biosensors were treated with αS PFF or monomer (10 nM), and FRET was measured 24 h later. Knockdown of the VCP adaptor UBXD1 significantly increased FRET efficiency compared with scramble siRNA control, whereas application of monomeric αS did not alter the FRET signal (Fig. 7A). To confirm UBXD1 is an essential VCP cofactor for seeding, we knocked down UBXD1 using shRNA in primary HNs 5 days prior to αS PFF application. UBXD1 KD also increased phospho-αS staining compared with scrambled shRNA control (Fig. 7B-D). The increase in phospho-αS staining was associated with a minor change in cell viability that was measured by MTT assay after 10 (1.08 vs. 0.92 0, *p* = 0.0329) and 15 days (*p*= > 0.9999) in culture (Fig. S5).

**Fig. 8:**
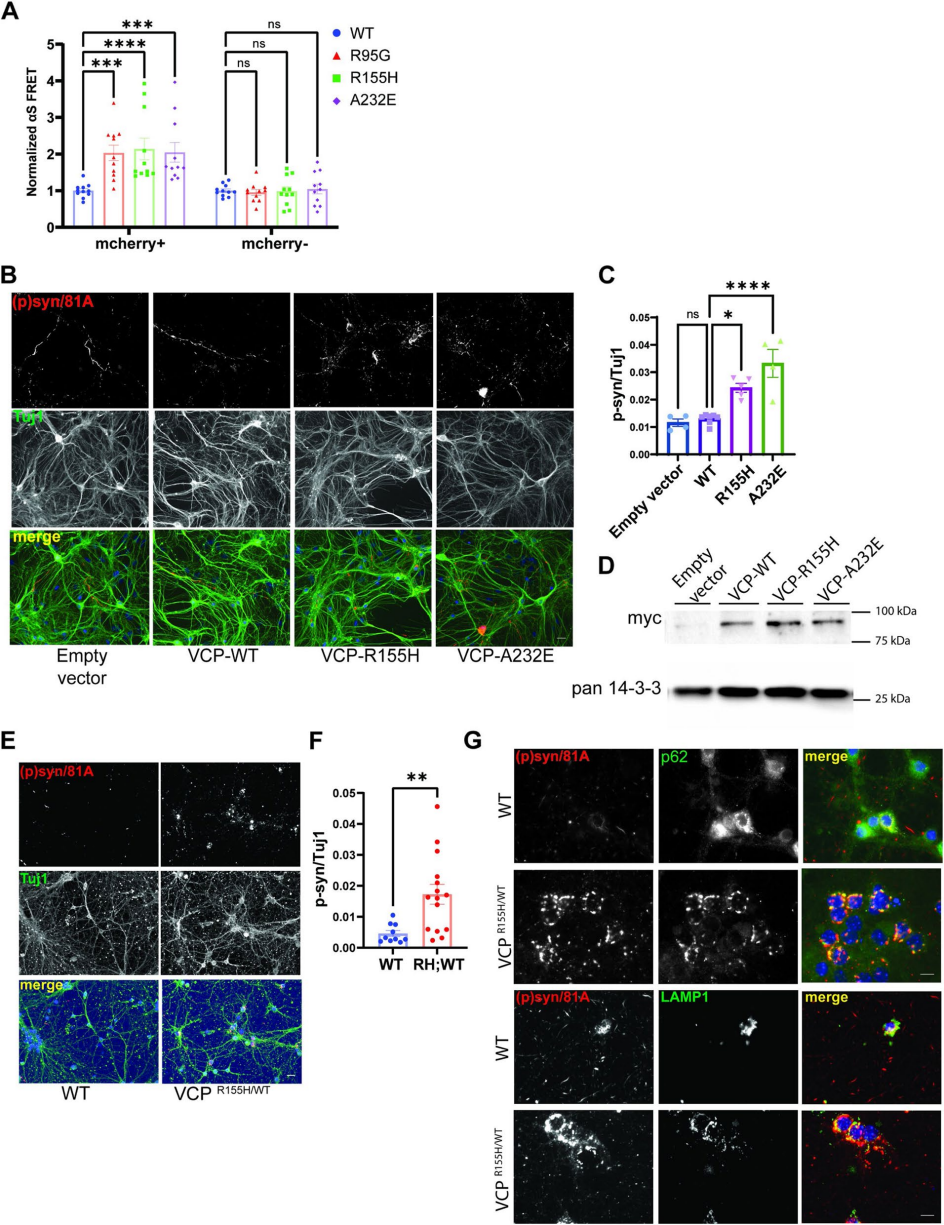
VCP disease mutation expression enhances αS seeding. **A** αS biosensors were transfected with plasmids expressing VCP-WT, or one of three disease mutations (R95G, R155H and A232E) fused to an mcherry tag for 24 hours and then treated with αS PFF(10nM). FRET efficiency is quantified in mCherry+ and mCherry- cells separately and all normalized to VCP-WT (n=11 repeats for each group. ***p < 0.001; ****p < 0.0001; ns, no significance; two-way ANOVA with Dunnett’s correction). **B** αS biosensors were transfected with plasmids expressing mCherry or mCherry-VCP-WT for 24 hours and then treated with αS PFF (10nM). FRET efficiency isquantified in mCherry+ and mCherry- cells separately and all normalized to mCherry. (n=11 repeats foreach group. ***p < 0.001; ****p < 0.0001; ns, no significance; two-way ANOVA with Dunnett’scorrection). **C** Immunofluorescence for phospho-αS and Tuj1 in HNs treated with empty control lentiviral vector, VCP-WT-myc or one of two myc tagged VCP disease mutations (R155H or A232E). HNs were transduced with lentivirus as indicated for 5 days before 10nM αS PFF treatment. HNs were harvested after another 5days. Scale bar=20μm. **D** Quantitation of phospho-αS/Tuj1 staining in 8C. Experiments were repeated from 3 different cultures. *p < 0.05; ****p < 0.0001; ns, no significance compared with the VCP-WT group by one-way ANOVA. **E** Immunoblot of lysates from HNs overexpressing lentiviruses expressing empty vector, VCP-WT-myc, VCP-R155H-myc or VCP-A232E-myc using an anti-myc antibody and pan 14-3-3 as a loading control. **F** Immunofluorescence for phospho-αS and Tuj1 in HNs from wild-type mice or mice carrying a VCP-R155H knock-in allele(VCP^R155H/WT^) treated with αS PFF (1μg/ml) for 5 days. **G** Quantitation of phospho-αS/Tuj1 staining in 8F. Neurons came from 10 and 15 independent cultures from WT and VCP^R155H/WT^ embryos. Outlier isremoved by ROUT method, Q=1%, followed by Student’s t test. p<0.0001), Scale bar =20μm. **H** Immunofluorescence of WT or VCP^R155H/WT^ HNs treated with αS PFF (1μg/ml) and harvested 5 days later with anti-phospho-αS (red) and p62 (green) (upper panels) or anti-phospho-αS (red) LAMP1 (green)(lower panels). Scale bar =10μm

To understand if VCP disease mutations augment αS seeding activity, we first evaluated αS seeding in the setting of mutant VCP overexpression. αS biosensors were transfected with mCherry-tagged VCP-WT or one of three different mCherry-tagged VCP disease mutations (R95G, R155H, and A232E) for 24 h then treated with αS PFF (10 nM) and quantified for FRET efficiency 24 h later. Transfected cells are selected for mCherry signal via flow cytometry simultaneously with FRET. mCherry positive cells increased FRET in VCP disease mutant expressing cells compared with VCP-WT control. Whereas cells not expressing mCherry were unchanged (Fig. 8A and S3B). Notably, transfection of αS biosensors with mCherry-tagged VCP-WT modestly increased FRET efficiency as compared with mCherry alone (Fig. 8B). To evaluate this in primary HNs, we transduced neurons with lentiviral constructs (CCIV) expressing wild-type VCP-myc or one of two different VCP disease mutations (VCP^R155H^-myc and VCP^A232E^-myc) or empty vector (CCIV), 5 days prior to αS PFF application. Lysates from similarly treated HNs demonstrated comparable levels of myc-tagged VCP-WT or mutant expression. Following 5 days, VCP-R155H and VCP-A232E expressing neuronal cultures had an increase in phospho-αS staining compared with control and VCP-WT expressing cultures (Fig. 8C-E).

In order to explore αS PFF seeding in a non-overexpressed neuronal system, we cultured primary HNs from VCP^WT/WT^ and VCP^RH/WT^ embryos, treated them with αS PFF, and then immunostained for phospho-αS 5 days later. VCP-R155H mutation knockin mice have been previously generated and characterized [42]. VCP^RH/WT^ HNs had a significant increase in phospho-αS staining compared with VCP^WT/WT^ HN controls (Fig. 8F-G). Notably, the phospho-αS staining in VCP^RH/WT^ treated HNs was less filamentous and predominantly perinuclear compared to VCP^WT/WT^ HNs. The perinuclear phospho-αS aggregates co-localized with SQSTM1 and LAMP1 suggesting an alteration in aggregate trafficking (Fig. 8H).

### VCP disease mutation expression increases αS seeding in vivo

We next examined the effect of pathogenic VCP mutations on αS seeding in vivo. VCP^RH/WT^ mice display no neuronal loss, TDP-43 inclusions, or pathologic features consistent with autophago-lysosomal dysfunction up to 13 months old [43]. To explore an additional VCP mouse model that only expresses a VCP disease mutant allele, we also used a mouse line that deletes the VCP-WT allele and only allows expression of a single VCP-R155C mutant allele following tamoxifen treatment (VCP^R155C/FL^; Rosa26-Cre ERT2 (cVCP-RC) (Fig. S6) [43]. Similar to our previous study, lysates from the cortex of VCP^RH/WT^ mice have no changes in the levels of autophagic proteins, SQSTM1, ER stress (BiP/GRP78), or ubiquitinated proteins (Fig. S6B-C). Following 5 days i.p. tamoxifen treatment, cortical lysates from cVCP-RC mice have a 41% reduction in total VCP protein level but no changes in autophagic levels, SQSTM1, ER stress (BIP/GRP78), or ubiquitinated proteins (Fig. S6B-C). However, high molecular weight ubiquitinated proteins and SQSTM1 levels increase with age, as demonstrated by immunoblot of cortical lysates at 6 months after tamoxifen, supporting that VCP dysfunction is present (Fig. S6D-E). We have previously demonstrated that an increase in Gal3 levels occurs early, before autophagic dysfunction in VCP^RH/WT^ mouse muscle [17]. Similar to skeletal muscle, Gal3 and LAMP1 levels are increased in both VCP^RH/WT^ and cVCP-RC mouse cortical lysates suggesting an accumulation of damaged late endosomes (Fig. S6B-C) [15, 17].

**Fig. 9:**
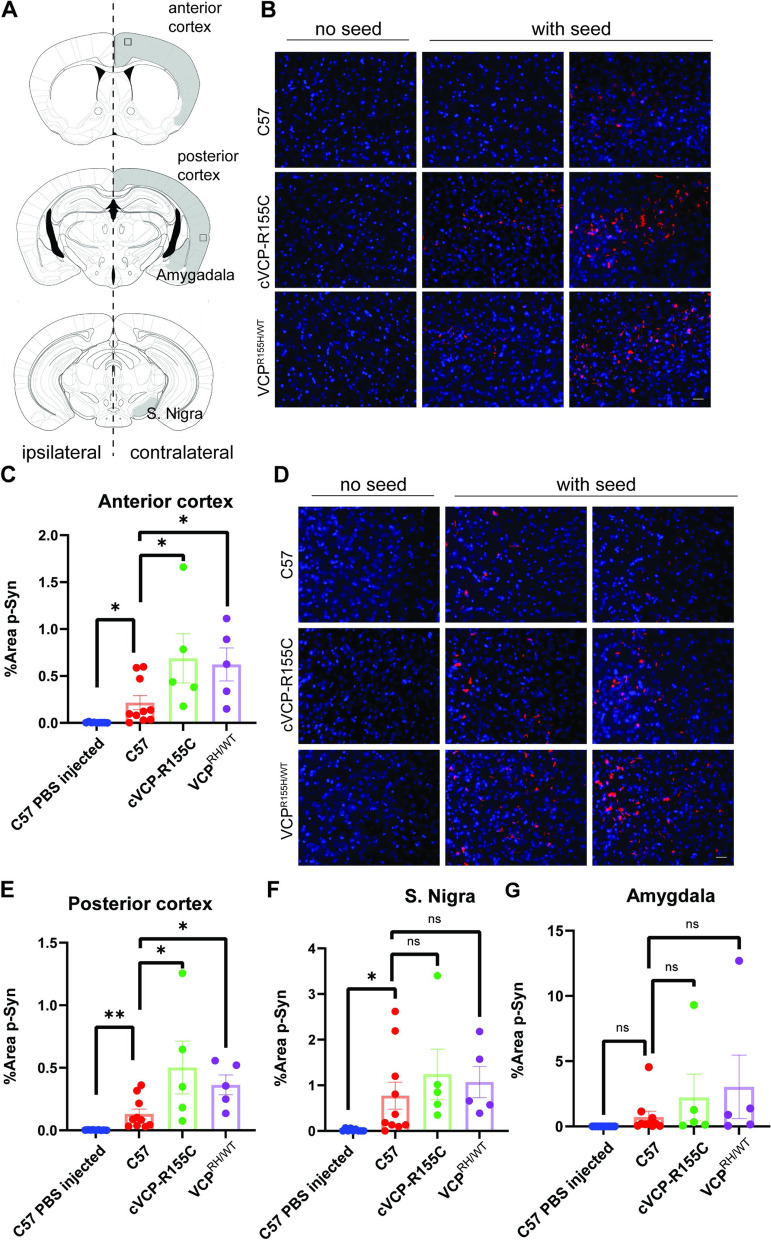
VCP disease mutations enhance αS seeding *in vivo*. **A** Drawing of coronal sections through mouse brains. Shaded regions indicate areas utilized for quantitation and boxes denote regions corresponding to the represntative images in B and D. **B** Representative immunofluorescence images with pSer129-syn antibody (red) of anterior cortices from C57, cVCP-R155C, and VCPR155H/WT mice injected unilaterally into the striatum with 5ug αS PFF after 90 days. 4 month aged C57 (*n*=10), VCP R155H/WT (*n*=5) and VCP R155C/FL; Rosa26-Cre ERT2 (cVCP-R155C) (*n*=5). Scale bar=25 μm. **C** Quantitation of the percentage of p-syn in entire anterior cortices (* *P*<0.05 by student’s t-test). **D** Representative immunofluorescence images with pSer129-syn antibody of posterior cortices from C57, cVCP-R155C, and VCPR155H/WT mice as described in A. scale bar=25 μm. **(E)** Quantitation of the percentage of p-syn in posterior cortices (* *P*<0.05, **P <0.01(* *P*<0.05 by student’s t-test). **F-G** Quantitation of p-syn in Amygdala and substantia Nigra. (ns, no significance student’s t-test)

We injected 5μg αS PFF or PBS into the striatum of 4-month-old C57 control, VCP^RH/WT^, or cVCP-RC mice and harvested the brain after 3 months (Fig. 9A). Age-matched untreated control, VCP^RH/WT^, cVCP-RC, C57 mice, or mice treated with PBS had no phospho-αS staining in any brain regions (Fig. 9A-E). In contrast, C57 control mice injected with αS PFF had a significant increase of phospho-αS in multiple brain regions (Fig. 9A-E). We found phospho-αS staining was significantly increased in the anterior and posterior cortices of VCP^RH/WT^, and cVCP-RC injected with αS PFF compared with that of C57 (Fig. 9A-E). Other brain regions such as the amygdala and substantia nigra trended toward an increase in phospho-αS staining but did not reach statistical significance (Fig. 9F-G).

### VCP disease mutations enhance TDP-43 seeding

**Fig. 10:**
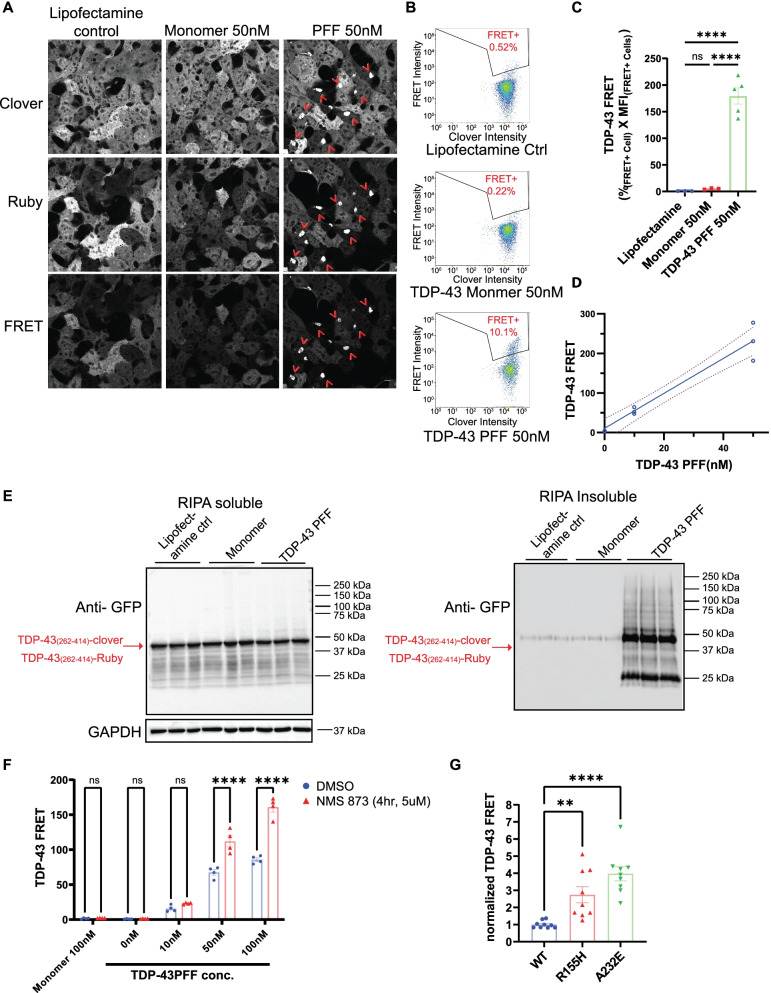
VCP inhibition or VCP disease mutations enhance TDP-43 seeding in cells. **A** Representative FRET confocal microscopy images of TDP-43 biosensor line (TDP-43 Clover/Ruby) treated with empty Lipofectamine (left, ctrl), 50nM TDP-43 monomer (middle) and 50nM TDP-43 PFF (right) after 48 hours. Scale bar= 10μm. **B** Tracing of FRET signal via flow cytometry. FRET+ gate (Clover vs FRET) was drawn from empty Lipofectamine treated cells with no aggregation. **C** Quantitation of integrated FRET signal is measured by % FRET+ cell * Median Fluorescent Intensity (FRET+ cells). **** *p* < 0.0001, n.s., no significance by one-way ANOVA. error bars are ±S.E.M. **D** Graph of FRET efficiency from TDP-43 biosensors were treated with TDP-43 PFF at different concentration and harvested after 48 hours (each dot represents triplicates in each condition). **E** Immunoblot for TDP-43 CTF (anti-GFP) from detergent soluble and insoluble lysates of TDP-43biosensor cells treated with TDP-43 monomer or PFF and then harvested after 48 hours. Note that the RIPA insoluble fraction accumulated high molecular weight TDP-43 positive multimers. GAPDH is a loading control. **F** Normalized FRET from TDP-43 biosensors co-treated with TDP-43 monomer or PFF and NMS-873 (5μm) or DMSO vehicle control for four hours followed by washout. Cells were harvested at 48 hours after the treatment, and analyzed the same as 10C. FRET signal is normalized to DMSO 100nM PFF treated group. (*n* >4 repeats for each group. ****p < 0.0001; n.s. no significance, two-way ANOVA with Šidák correction) **G** TDP-43 biosensors were transfected with plasmids expressing VCP-WT, or one of two disease mutations (R155H and A232E) for 24 hours and then treated with TDP-43 PFF (10nM) for 48 hours. FRET efficiency is all normalized by VCP^WT^. (*n*=9 repeats for each group. *p <0.05, **p < 0.01; ****p < 0.0001; one-way ANOVA with Dunnett’s correction)

A subset of VCP patients have Parkinsonism and post-mortem evidence of αS pathology. However, most VCP patients have TDP-43 inclusions in the CNS and muscle [8–10]. To evaluate the role of VCP in the seeding of TDP-43, we utilized a HEK TDP-43 FRET biosensor that expresses both a Clover tagged-TDP-43 C-terminal fragment (CTF) and Ruby tagged-TDP-43 CTF (aa 262-414) (TDP biosensors). Exogenously applied preformed fibrillar TDP-43 (TDP-43 PFF 50 nM) with Lipofectamine, but not monomeric TDP-43 (50 nM), recruited the aggregation of soluble intracellular TDP-43 CTF after 48 h. This resulted in Clover and Ruby positive aggregation as visualized by fluorescence (Fig. 10A). Similar to the αS biosensor line, TDP-43 PFF-dependent aggregates can also be detected by FRET under confocal microscopy (excitation = 488 nM and emission = 605-625 nM) (Fig. 10A). Cells with FRET were quantified using a Clover vs. FRET plot with flow cytometry and selected against empty Lipofectamine treated control with no aggregates (black polygon gates in Fig. 10B). FRET efficiency is calculated as a percentage _(FRET positive cell)_* MFI _(FRET positive cell)_ [29]. The TDP-43 FRET signal is TDP-43 PFF-dependent since little signal was captured in monomer and empty Lipofectamine treated groups (Fig. 10A-C). In addition, this FRET signal is sensitive and quantitative in a concentration-dependent manner (Fig. 10D). To biochemically confirm that the FRET signal corresponded to TDP-43 CTF aggregation, we analyzed the cell lysates by fractionation immunoblot. An anti-GFP antibody was used to detect Clover- and Ruby-tagged TDP-43 CTF (~ 40 kDa). Only TDP-43 PFF treated TDP biosensor lines generated detergent-insoluble and high molecular weight TDP-43 CTFs (Fig. 10E).

As with αS-biosensors, co-application of the VCP inhibitor NMS-873 (5uM) for 4 hours at the time of seed application (50 and 100 nM) to TDP biosensors followed by washout significantly increased FRET as compared with DMSO treated control cells 2 days later (Fig. 10F). This phenomenon is seed-dependent since NMS-873 failed to increase FRET in TDP-43 biosensors when TDP-43 monomer treatment was used. To explore the role of VCP disease mutations on TDP-43 seeding, we transfected TDP biosensors with myc tagged-VCP-WT or one of two different VCP disease mutations (R155H and A232E) for 24 h, followed by TDP-43 PFF application. Cells expressing either VCP disease mutations exhibited an increase in FRET efficiency 48 h later compared with VCP-WT control (Fig. 10G and S7).

**Fig. 11:**
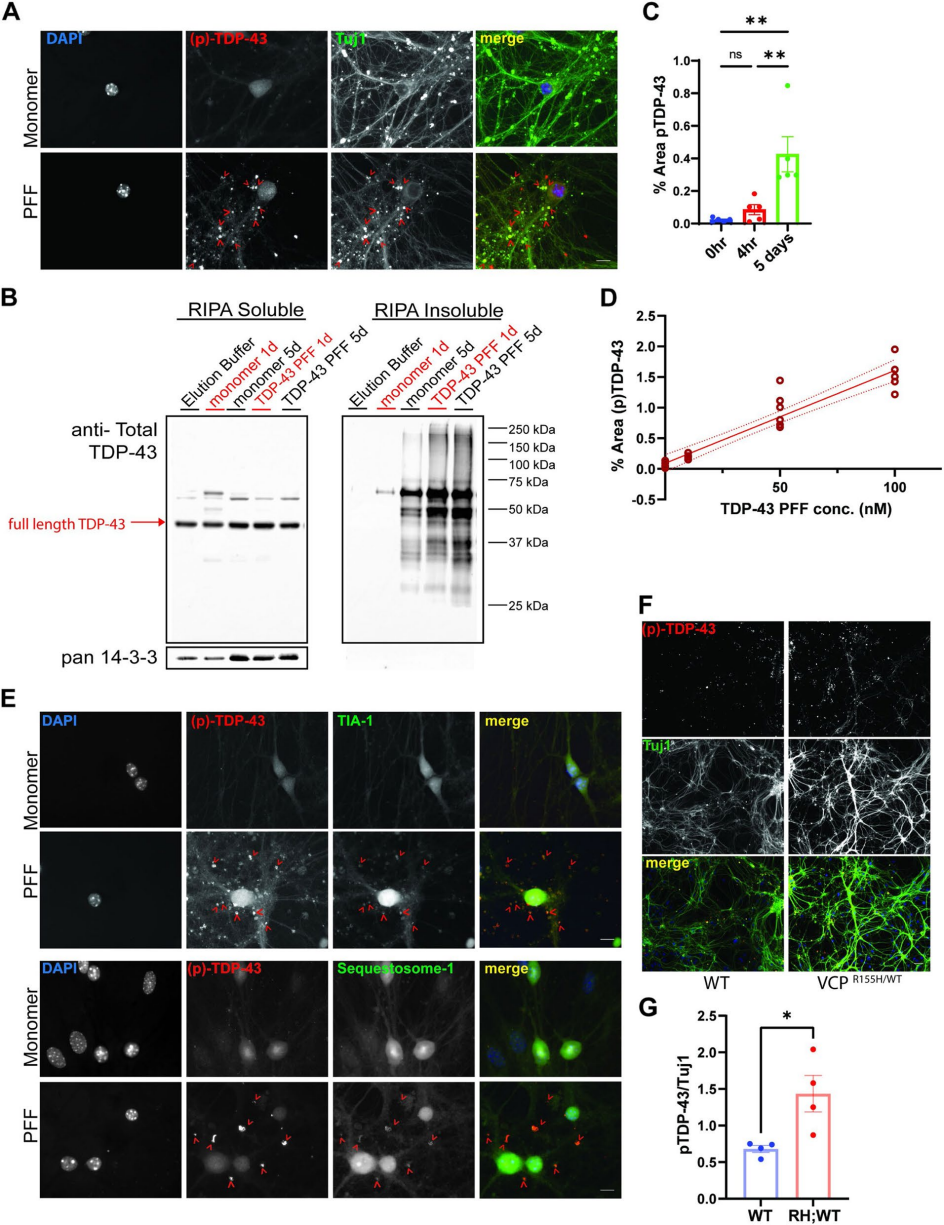
VCP disease mutations enhance TDP-43 seeding in neurons. **A** Immunofluorescent staining for pTDP-43 (red), Tuj1 (green), and nuclei (blue) in primary hippocampal neurons treated with 10nM TDP-43 monomer or 10nM TDP-43 PFF for 5 days. Scare bar=10μm. **B** Immunoblot for TDP-43 from detergent soluble and insoluble lysates of HNs treated with 10nM TDP-43 monomer or 10 nM PFF and then harvested after 1 or 5 days. Note that the RIPA insoluble fraction has high molecular weight TDP-43 positive multimers. pan 14-3-3 is a loading control. **C** Quantitation of area of pTDP-43 immunofluorescence in HNs after 4 hours or 5 days after 10nM TDP-43 PFF treatment. **D** Quantitation of area of pTDP-43 immunofluorescence treated with the indicated concentrations of TDP-43 PFF and harvested at 5 days. **E** Immunofluorescent images for pTDP-43, SQSTM1, and TIA-1 from neurons treated with TDP-43 monomer or TDP-43 PFF for five days. Scare bar=10 μm. **F** Immunofluorescence for phospho-TDP-43 (red) and Tuj1 (green) in HNs from wild-type mice or mice carrying a VCP-R155H knockin allele (VCPR155H/WT) treated with TDP-43 for PFF for 5 days. (Scare bar= 20μm). **G** Quantitation of phospho-TDP/Tuj1 staining (Neurons coming from 3 and 4 independent cultures from WT and VCP^R155H/WT^ embryos. Outlier is removed by ROUT method, Q=1%, followed by Student’s t test. *n*=4, WT and VCP^R155H/WT^ group respectively. *p<0.05.)

To further explore the effect of TDP-43 seeding in a more relevant system, we developed a TDP-43 seeding assay in primary HNs. The addition of TDP-43 PFF (10 nM) to HNs resulted in the appearance of phosphorylated TDP-43 Ser409/410 (pTDP) positive puncta (Fig. 11A). Fractionation of lysates from HNs one or 5 days post-treatment with buffer, monomeric TDP-43 (10 nM), or TDP-43 PFF (10 nM) and subsequent immunoblot for TDP-43 revealed an increase in high molecular weight TDP-43 in the RIPA insoluble fraction of TDP-43 PFF treated HNs (Fig. 11B). The pTDP staining was not present immediately after the addition of TDP-43 PFF since no increase in pTDP above that seen prior to seed application. In contrast there was a significant increase in pTDP staining when TDP-43 PFF treated HNs were stained after 5 days (Fig. 11C). Morever, the amount of pTDP staining post-TDP-43 PFF treatment correlated with the concentration of TDP-43 PFF seed used (Fig. 11D). In addition, TDP-43 PFF induced cytosolic pTDP-43 puncta that co-localized with TIA1 and SQSTM1 similar to pathologic TDP-43 inclusions in patients (Fig. 11E) [44]. As seen with VCP mutant expression in TDP-43 biosensors, treatment of primary HNs from VCP^WT/WT^ and VCP^RH/WT^ embryos with TDP-43 PFF (10 nM) revealed an increase pTDP-43 puncta in VCP^RH/WT^ HNs compared with VCP^WT/WT^ HNs (Fig. 11F-G).

## Discussion

Functional genomic screens utilizing CRISPR knockout approaches are an invaluable tool to elucidate proteins related to distinct cellular pathways. Here, we employed a CRISPR whole-genome KO screen to identify modifiers of αS seeding using an αS biosensor cell line. αS seeding spans many cellular processes that include endocytic uptake, vesicular trafficking, templated aggregate conversion, and protein degradation by both the proteasome and autophagy. Our screen identified proteins associated with vesicular trafficking between the ER, Golgi and endosome, and the cellular stress response. Notably, these pathways have been identified as modifiers of αS toxicity and stability in yeast and cell models [45, 46]. Vesicular trafficking may have been particularly enriched since our screen and the αS biosensor cell line required αS PFFs to be applied with the carrier, Lipofectamine.

Our further studies expanded upon the role of VCP in αS seeding. VCP is a multifunctional protein necessary for many ubiquitin-dependent processes, including protein degradation, vesicle trafficking, cell division, and organelle clearance [7]. Recently, we identified a role for VCP in recognition of permeabilized late endosomes and their subsequent lysophagic degradation [15]. Endocytosed proteopathic seeds such as αS and Tau enter the cytoplasm, where templated aggregate conversion of soluble monomer occurs by damaging the endosomal membrane [5, 15, 47]. The fate of permeabilized late endosomes depends upon the degree of membrane damage. For example, some damaged endosomes are rapidly repaired by ESCRT proteins [48]. In contrast, endosomes damaged beyond repair are tagged by intracellular galectins such as galectin-3 [15]. Galectin positive endosomes recruit the ubiquitin ligase, Trim16, which ubiquitinates endosomal membrane proteins [49]. Notably, only lysine-63 linked ubiquitin chains on the endosome surface are targeted for lysophagy [15]. VCP, in association with UBXD1, PLAA, and the deubiquitinase YOD1 recognize and cleave lysine-48 linked ubiquitin chains on damaged late endosomes, leaving lysine-63 linked ubiquitin chains allowing lysophagic degradation [15]. Loss of VCP or VCP disease mutant expression leads to the persistence of galectin-3 positive damaged late endosomes in cells, mouse models, and patient tissue [15, 17].

A previous genomic screen using a Tau biosensor line identified several components of the ESCRT machinery as suppressors of Tau seeding [50]. Our screen identified VCP and Trim16 as suppressors of αS seeding. Further experiments found that knockdown of the VCP adaptor, UBXD1 that is necessary for lysophagy, and VCP disease mutations defective in lysophagy also increase αS seeding. One distinction between these two screens is the use of Lipofectamine to facilitate entry into the endocytic pathway of αS. Lipofectamine is known to damage endosomal membranes and may allow αS to generate larger “holes” that are not repaired by ESCRTS [48]. However, VCP’s role in seeding was not exclusively Lipofectamine dependent since VCP inhibition, knockdown, or VCP mutant expression facilitated “naked” αS PFF seeding in HNs and in vivo.

Our data supports a role for VCP in the surveillance of seed induced endosomal damage at the point of “endolysosomal escape”. Specifically, VCP inhibition for 4 hours at the time of seed application is sufficient to enhance seeding in αS biosensors or HNs. This is similar to treatment with the lysomotropic agent, LLOMe, which enhances proteopathic seeding via increasing endolysosomal escape [5]. However, VCP’s participation in multiple cellular processes such as protein aggregate formation, proteasomal degradation and autophagy could also play a critical role in αS seeding. Future studies that track the entry and escape of endocytosed αS at the cell biologic level will help to clarify the role or roles for VCP in proteopathic seeding.

VCP disease mutations cause multisystem proteinopathy (MSP) [51]. MSP is a late-onset degenerative disorder with varied phenotypes and pathologies. These include inclusion body myopathy, ALS, and FTD [18]. While the predominant aggregate pathology in the brain is reported to be TDP-43, several studies support the identification of αS positive aggregates in the brain [8–10]. Indeed, ~ 5% of MSP patients have coincident Parkinsonism [19]. Notably, two recently identified families with a VCP-D395G mutation were found to have distinctive tau pathology leading to the description of a vacuolar tauopathy in the CNS [11]. Aggregate pathology in the skeletal muscle can be varied and include TDP-43, hnRNPA1/A2B1, SQSTM1, β-amyloid, desmin, and VCP [52–54]. Weakness typically precedes the onset of neurodegenerative features such as dementia by 10 years suggesting that pathology begins in peripheral tissue such as skeletal muscle [19]. Whether protein aggregates from skeletal can seed the aggregate process in motor neurons or cortical neurons, remain speculative. It is noteworthy that mice carrying the D395G missense mutation in VCP had an increase in Tau seeding, supporting that VCP disease mutations can facilitate the propagation of different aggregate species [11].

TDP-43 inclusions are a prominent feature in affected VCP disease tissue [9, 52]. Our data further support that TDP-43 PFF similar to αS-PFF can seed pathologic TDP-43 inclusions in HNs. TDP-43 seeding in HNs recapitulates several features of TDP-43 pathology such as phosphorylation, cytoplasmic redistribution, and co-localization with stress granule markers and SQSTM1 [55]. VCP disease mutation expression increases seeding associated pathologic TDP-43 inclusions as measured by increased phospho-TDP-43 immunostaining in HNs. This process is TDP-43 PFF dependent since monomeric TDP-43 fails to have the same effect. Whether the accumulation of TDP-43 inclusions is also affected by an additional role for VCP in stress granule clearance remains to be determined [56].

Halting or mitigating the pathologic spread of protein inclusions in neurodegenerative disorders such as Parkinson’s disease, ALS and fronto-temporal dementia has potential therapeutic implications. Our study identifies a VCP dependent endocytic pathway that suppresses the proteopathic spread of αS in vivo and TDP-43 in vitro. Future studies aimed at defining the molecular components and mechanism related to VCP associated aggregate seeding may lead to therapies targeting multiple proteinopathies and degenerative diseases.

## Conclusion

Proteins associated with neurodegeneration (e.g., TDP-43, αS, and tau) can behave as proteopathic seeds leading to protein inclusions and neuronal death. Utilizing CRISPR whole-genome screening, this study identifies suppressors of αS seeding that include the neurodegeneration-associated protein, VCP. VCP inhibition or knockdown enhances αS seeding, independent of VCP’s established roles in ER stress, autophagy, and the ubiquitin proteasome system. VCP is essential for the lyosophagic degradation of permeabilized late endosomes. Enhancing lysosomal membrane permeability with the small molecule, LLoMe, increases αS seeding. VCP inhibition or knockdown similarly increases αS seeding. Whether this is due to VCP’s role in regulating the lysophagic degradation of damaged late endosomes or another VCP dependent role remains speculative. However, the fact that knockdown of the lysophagy co-factor UBXD1 or expression of VCP disease mutations defective in lysophagy also increase αS seeding suggests that VCP secondarily protects cells and neurons from the endolysosomal escape of proteopathic seeds. VCP mutations have a similar effect on αS and TDP-43 seeding. This may explain the phenotypic variability of VCP-MSP pathologies. Our study supports a unified model of proteopathic seeding in which a common VCP dependent cellular mechanism supports the propagation of distinct aggregate species.

## Supplementary Information

**Additional file 1.**

**Additional file 2.**

**Additional file 3.**

**Additional file 4.**

## Data Availability

The datasets generated and/or analyzed in this study are available from the corresponding author Conrad Weihl on request.

## Abbreviations

3-MA: 3-Methyladenine
αS: Alpha-synuclein
ALS: Amyotrophic lateral sclerosis
Eer I: Eeyarestatin I
EGCG: Epigallocatechin Gallate
FRET: Förster resonance energy transfer
FTD: Frontotemporal Dementia
GARP: Golgi-associated retrograde protein complex
gRNA: Guide RNA
HNs: Hippocampal Neurons
LLoMe: L-leucyl-L-leucine methyl ester
MSP: Multisystem proteinopathy
NGS: next generation sequencing
PFF: Pre-formed fibril
TDP-43: TAR DNA binding protein 43
VCP: Valosin Containing protein
cVCP-RC: VCPRC/FL; Rosa26-Cre ERT2
