# Supplementary material for: VCP suppresses proteopathic seeding in neurons
Source: Mol Neurodegener. 2022 Apr 12;17:30. doi: 10.1186/s13024-022-00532-0 (PMC9004082; doi:10.1186/s13024-022-00532-0)
Supplement: Supplementary file 1 — Additional file 1. [file 13024_2022_532_MOESM1_ESM.pdf]

A

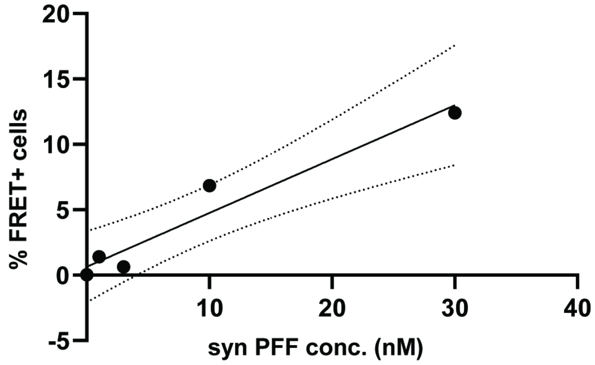

Figure S1: <u>spCas9-gRNA αS biosensor maintain seeding capacity.</u> spCas9 αS biosensor was treated with pooled library and maintained as described in Fig 2A, followed by αS seeding at different concentration. After 24hours, cells are harvested and the percentage of FRET is measured by flow cytometry.

A

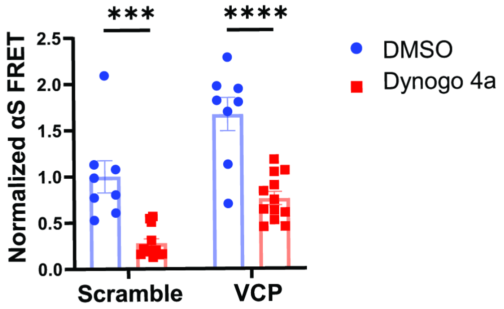

Figure S2: Dynogo 4A inhibits VCP dependent increased seeding in αS biosensor.αS biosensors with VCP or scramble siRNA was treated with Dynamin inhibitor Dynogo4a or DMSO the same as Figure 3B and harvested for flow cytometry. (n≥ 8 repeats for each group. ***$p < 0.001$; ****$p < 0.0001$; two-way ANOVA with Šídák's multiple comparisons test).

A

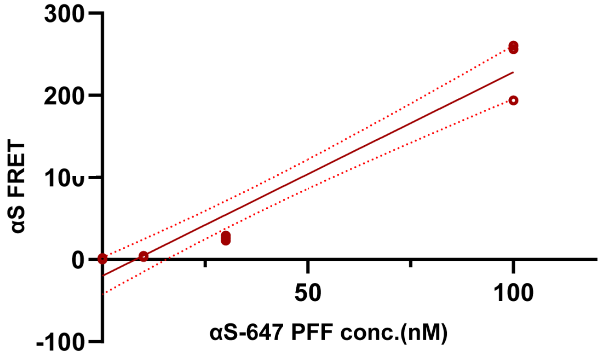

Figure S3: <u>αS biosensor shows seeding activities in a concentration dependent manner.</u> Alexa647 tagged αS PFF show moderate seeding capacity according to FRET assay.

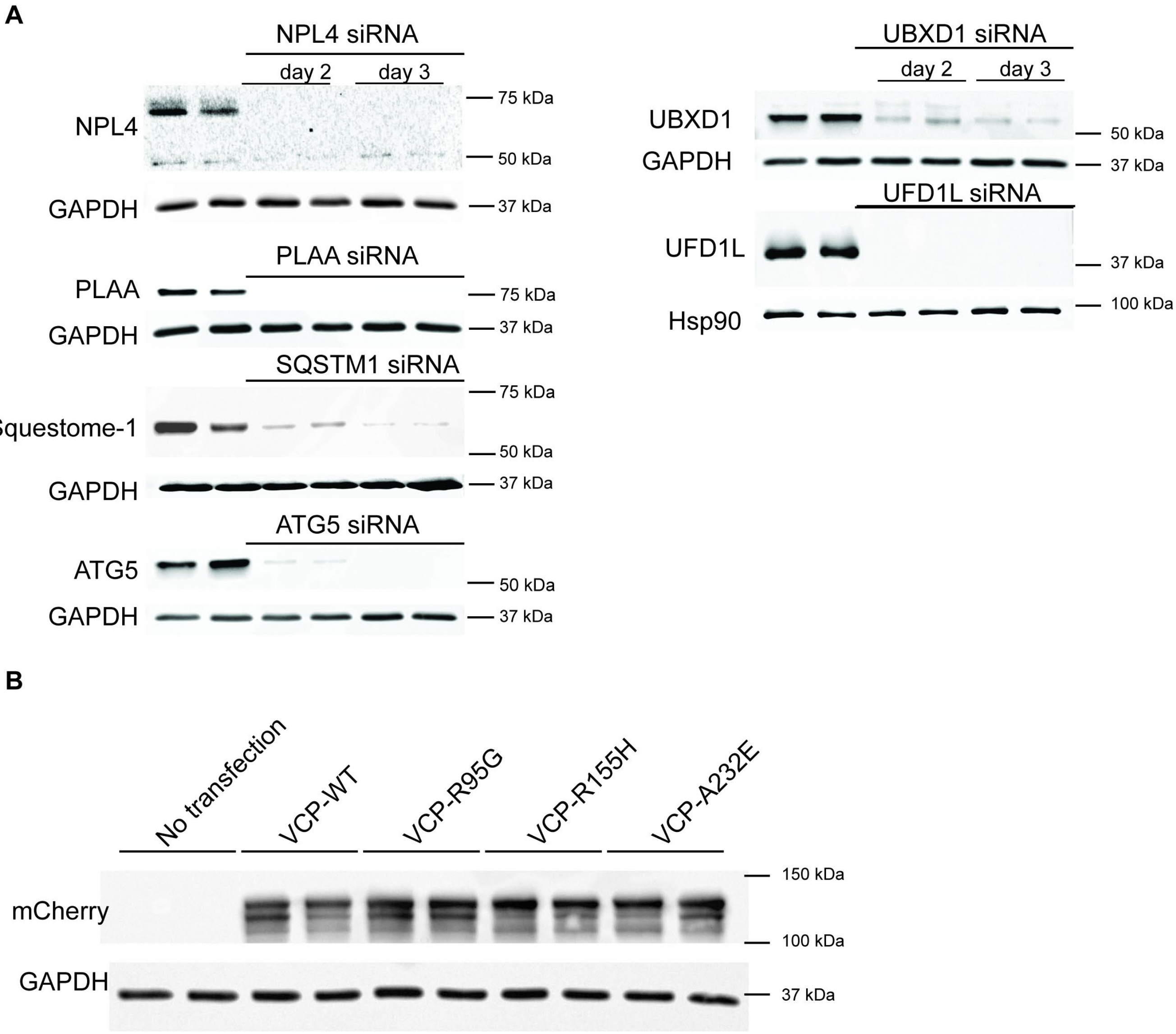

Figure S4: Gene knockdown efficiency and VCP vector transfection. **(A)** αS biosensor is reverse transfected with siRNA (6pmol). Cell lysis are saved at 48 and 72 hours after transfection. Immunoblot is preformed on each KD for protein level change. **(B)** immunoblot with anti-mCherry of αS biosensor lysate with overexpression of different VCP vectors.

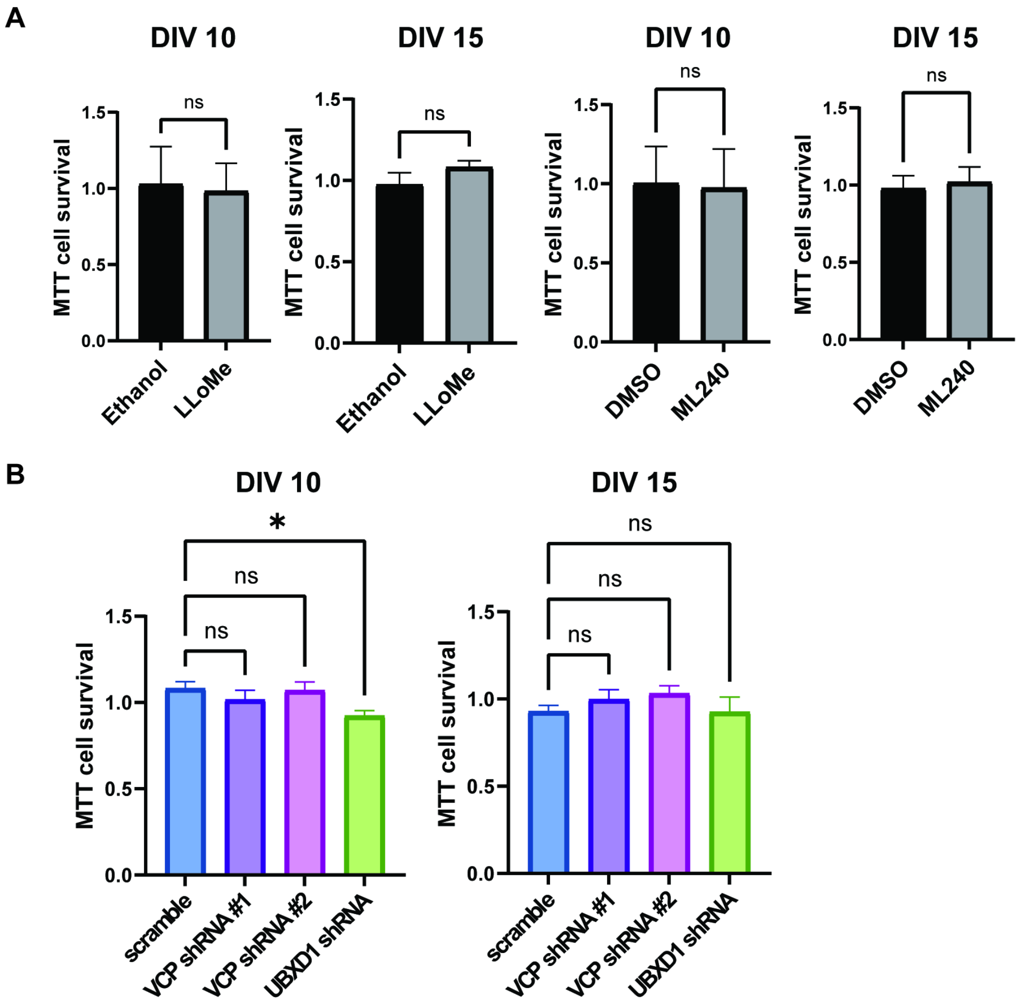

Figure S5: Cell viability in HNs with treatment.
(A) HNs are treated with LLoMe (1uM) or ML240 (100nM) for 4 hours at DIV10. Cell are tested by MTT assay right after the temporal treatment (DIV10) or at the time we harvest the cell for staining (DIV15).
(B) HNs are transduced with lentivirus shRNA targeting genes as indicated at DIV4 (MOI=10). Cell viabilities are tested by MTT assay at DIV10 and DIV15 respectively. p<0.05 and n.s.=no significant by one-way ANOVA.

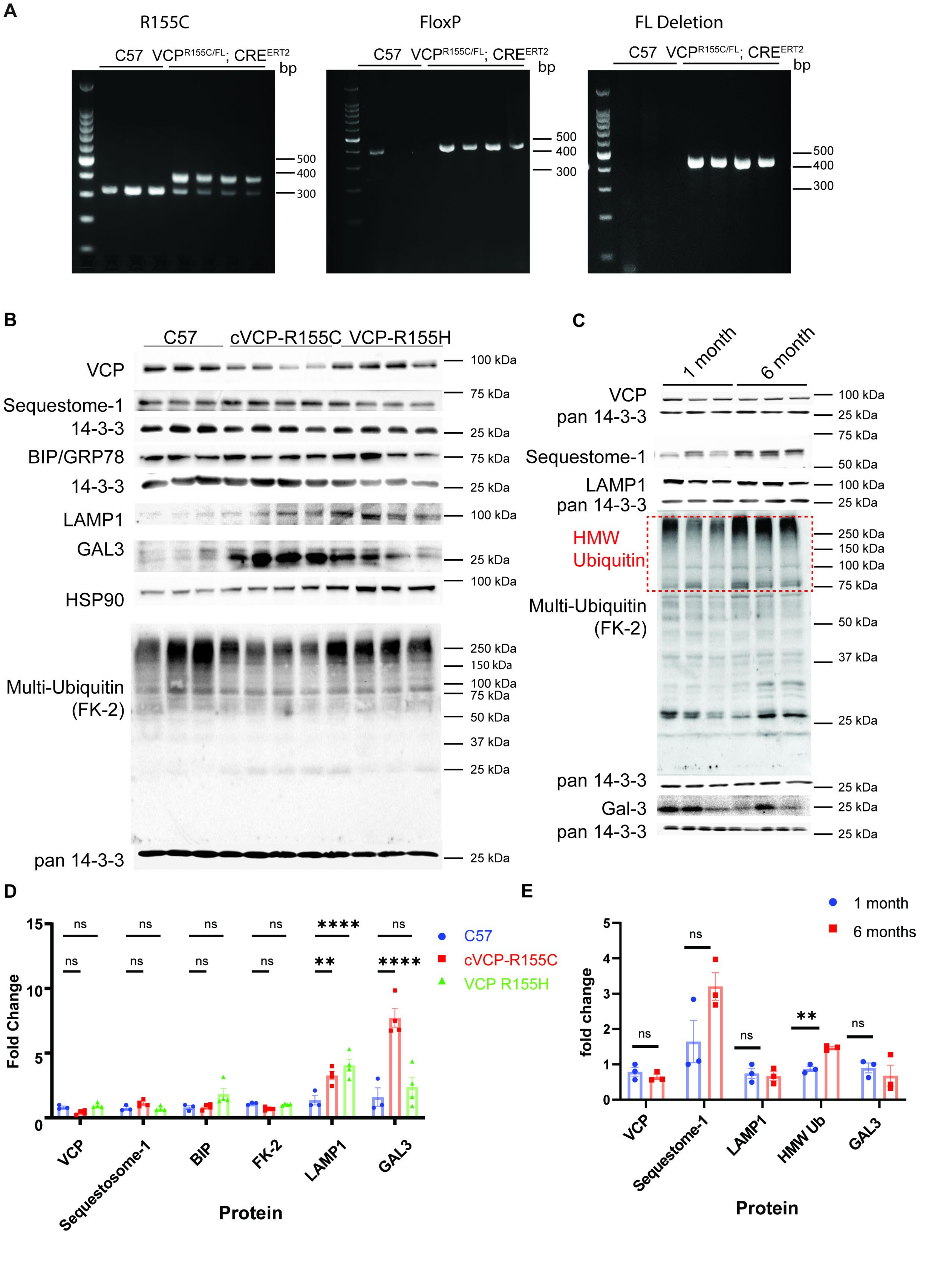
A
R155C
FloxP
FL Deletion
C57 VCPR155C/FL; CREERT2
bp
500
400
300
B
C57 cVCP-R155C VCP-R155H
VCP
Sequestome-1
14-3-3
BIP/GRP78
LAMP1
GAL3
HSP90
Multi-Ubiquitin (FK-2)
pan 14-3-3
100 kDa
75 kDa
25 kDa
250 kDa
150 kDa
50 kDa
37 kDa
C
1 month
6 month
HMW Ubiquitin
Gal-3
D
Fold Change
C57
cVCP-R155C
VCP R155H
ns
**
****
VCP
Sequestosome-1
BIP
FK-2
LAMP1
GAL3
Protein
E
fold change
1 month
6 months
HMW Ub

Figure S6: VCP disease mutant mice accumulate Galectin-3.
(A) Genotyping of cVCP-R155C mice. Cortical tissues from 4-month-old cVCP-R155C and C57 are genotyped. cVCP-R155C mice are i.p. injected with Tamoxifen in 5 continuous day and harvested after one month for genotyping. **(B)** Representative immunoblot for VCP, p62, pan 14-3-3, BiP/GRP78, Lamp1, Galectin-3, HSP90, and ubiquitin (FK2) from cortical brain lysates of 4-month-old C57 (n=3), $VCP^{R155H/WT}$ (n=4) and cVCP-R155C (n=4) mice. In the case of cVCP-R155C mice, they are intraperitoneally injected with tamoxifen at 90 days of age and the brain was collected after one month. **(C)** Quantification of band intensities of VCP, p62, Bip/GRP78, LC3, FK2, Lamp1 and Gal3. **(D)** Representative immunoblot for VCP, p62, 14-3-3, Lamp1, Galectin-3, and ubiquitin (FK2) from cortical brain lysates of cVCP-R155C mice following one month or six months of i.p. tamoxifen treatment. **(E)** Quantitation of band intensities of VCP, p62, Lamp1, FK2 and Gal3 (n=3 for both groups, multiple t test, p=0.001516, n.s.= no significance).

A

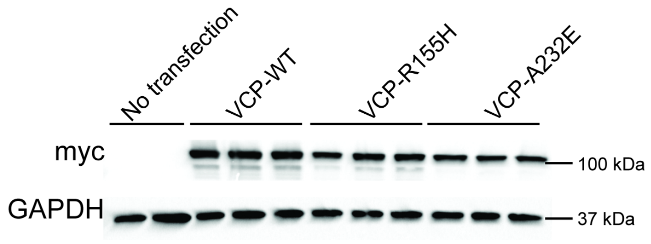

Figure S7: Immunoblot of VCP overexpression in TDP biosensor line.
Immunoblot of lysates from TDP biosensor overexpressing empty vector, VCP-WT-myc, VCP-R155H-myc or VCP-A232E-myc using an anti-myc antibody or GAPDH as a loading control.
